# Fractional dynamics study: analytical solutions of modified Kordeweg-de Vries equation and coupled Burger’s equations using Aboodh transform

**DOI:** 10.1038/s41598-024-61972-w

**Published:** 2024-06-03

**Authors:** Naveed Iqbal, Shah Hussain, Amjad E. Hamza, Ali Abdullah, Wael W. Mohammed, Mohammad Yar

**Affiliations:** 1 Deparment of Mathematics, College of Science, University of Ha’il, Ha’il, 2440 Saudi Arabia; 2 School of Science and Technology, University of Management and Technology, Sialkot Campus, Sialkot, 51310 Pakistan; 3 Department of Mathematics, Faculty of Science, Mansoura University, Mansoura, 35516 Egypt; 4 Department of Mathematics, Kabul Polytechnic University, Kabul, Afghanistan

**Keywords:** Aboodh residual power series method, Modified Kordeweg-de Vries equation (mKdV), Coupled Burger’s equations, Aboodh transform iteration method, Caputo operator, Applied mathematics, Computational science

## Abstract

The study examines the using of Aboodh residual power series method and the Aboodh transform iteration method (ATIM) to analyze modified Korteweg-de Vries equation (mKdV) beside coupled Burger’s equations in the framework of the Caputo operator. These sets of equations represent the non-linear wave description for various physical systems. Through APM and ATIM, the solution for the coupled Burger’s equations and the mKdV equation get accurate dynamics information that will reveal the nature of their interactions. Using mathematically proven techniques and computational simulations, the developed methods’ efficiency and reliability are illustrated in the complex behaviors of these nonlinear wave equations, so that we can gain deeper insights into their complex dynamics. The research is aimed at an increase of the knowledge about the fractional calculus utilization for nonlinear wave motion and it also provides analytical tools for an analysis of wave acting in different scientific and engineering areas.

## Introduction

Nonlinear partial differential equations (PDEs), involving complex physical phenomena in many areas of science and being one of the most important mathematical instruments of modelling, are the foundation of many studies. The function is typically not linear, and this behaviour is an analog for the complex connections of the variables. The system of nonlinear partial differential equations which represents such a system usually involve several unknown functions as well as their derivatives^1–4^. Such models are ubiquitously employed across physics, engineering, biology, and other branches of science, to explore properties of waves, fluids, patterns, and their interactions with populations. The conduct of nonlinear systems and getting the best results out of them means the use of new approaches and the latest computer tools. Unlike linear systems that can be simply constructed, solving and understanding nonlinear systems is always a complicated task^5–12^.

An important new area of mathematical modelling has emerged from the combination of fractional calculus and nonlinear partial differential equations (PDEs), providing a powerful perspective through which to understand complicated events with memory and non-local behaviours. Nonlinear partial differential equations (FPDEs) include nonlinear components and fractional derivatives, allowing non-integer order derivatives and nonlinear interactions to be described in systems^13–15^. For phenomena where memory effects, long-range interactions or anomalous diffusion are key, this combination has broad applications in many different areas, including physics, biology, engineering, and finance. The study of first-order partial differential equations (FPDEs) has attracted a lot of attention because, as compared to classical integer-order partial differential equations (PDEs), they can model complicated behaviours more accurately. However, these efforts greatly advance scientific knowledge and technological progress. This work starts with a detailed study of a particular class of fractional nonlinear partial differential equations, trying to find solutions that show the interesting complexity of these systems and to shed light on their special features^15–18^. The studies referenced cover a wide range of topics, from aerospace engineering to materials science and physics. Each one contributes valuable insights and advancements to its respective field. For instance, Shi et al.^19^ focus on predicting laminar-turbulent transition in aerodynamics, while Wang et al.^20^ propose an improved model for transonic boundary layers. On the materials science front, Hua et al.^21^ investigate void healing mechanisms in aeroengine steel, and Zhang et al.^22^ explore fatigue improvement in titanium alloy bolts. In physics, Zhu et al.^23,24^ present various studies on soliton solutions and nonlinear models using modified Schrodinger’s equations. These diverse studies highlight the breadth of research being conducted across different disciplines, each contributing to our understanding and technological advancements in their respective domains^25,26^.

For numerous reasons related to the most fundamental mathematical processes, the creation of novel travelling wave solutions to non-linear partial differential equations (NLPDEs) is of great importance. Geology, meteorology, biology, solid-state physics, fluid mechanics, chemical kinematics and chemical physics are just a few of the many scientific and technical fields that encounter the non-linear wave phenomena^27–30^. Significant non-linear wave phenomena such as diffusion, convection, dispersion, response, and dissipation are included in non-linear wave equations. Therefore, finding the precise answers to those equations has been a major focus of physics and mathematics. In search of precise solutions to these equations, several methods have been investigated and developed, including the generalised Kudryashov method^31^, the sine-Gordon expansion^32,33^, the Exp-function^34^, the perturbation^35,36^, the Lie symmetry^37^, the sins method^38^, the Ricatti equation expansion^39^, the improved tan($\varphi$/2)-expansion^40^, the Bernoulli sub-equation function^41^ and (G^′^/G)-expansion^42^.

A well-known model for nonlinear partial differential equations is the fractional KdV equation:

1 $$\begin{aligned} \begin{aligned}{}&D_{{\Im }}^{p} v(\zeta , {\Im })+6v(\zeta ,{\Im })\frac{\partial v(\zeta ,{\Im })}{\partial \zeta }+\frac{\partial ^{3} v(\zeta ,{\Im })}{\partial \zeta ^{3}}=0 \end{aligned} \end{aligned}$$

A variety of wave forms, including as those in ionized plasma, on a crystal lattice, as long internal waves in an ocean stratified by density, and in shallow waters with weak interactions, are described by the KdV equation.

The mKdV equation, in contrast, is a fractional modified Kordeweg-de Vries equation.

2 $$\begin{aligned} \begin{aligned}{}&D_{{\Im }}^{{\varrho }} v(\zeta , {\Im })+6v^{2}(\zeta ,{\Im })\frac{\partial v(\zeta ,{\Im })}{\partial \zeta }+\frac{\partial ^{3} v(\zeta ,{\Im })}{\partial \zeta ^{3}}=0 \end{aligned} \end{aligned}$$

Having the following initial condation

3 $$\begin{aligned} \begin{aligned} v(\zeta , 0)=W(\zeta ), \end{aligned} \end{aligned}$$

changed the course of soliton theory forever. The inverse scattering transform and an endless number of conservation laws for the KdV equation were both created using it, which in turn led to the discovery of the Lax pair for the KdV equation^43^. Various writers have tackled the topic of precise solutions using various approaches, including the Exp-function, the first integral, the tanh methods, Bifurcation, (G’/G)-expansion and many more^44–49^.

The Fitzpatrick-Thomas set of PDEs theory, Descontours al, the Burgers Equations^50–52^ describe the diffusion phenomenon nonlinearly. Viscous flow modeling, turbulence, and fluid mechanics are the major areas applied by Burgers equation^53^. Three-dimensional coupled Burgers models are used for scaled volume concentration in fluid suspensions. For sedimentation and evolution processes, the units are chosen accordingly. Along with others, in reference to the older editions, more details like coupled burgers equations are shown(^54,55^). In^56^, the Brooks and Engel equation was obtained using fractional derivative to be relevant to the new technique of fractional calculus.

With fractional derivatives, the system of nonlinear coupled Burger’s equations may be expressed as:

4 $$\begin{aligned} \begin{aligned}{}&D_{{\Im }}^{{\varrho }} v_{1}(\zeta , {\Im })-\frac{\partial ^2 v_{1}(\zeta ,{\Im })}{\partial \zeta ^2}-2v_{1}(\zeta ,{\Im })\frac{\partial v_{1}(\zeta ,{\Im })}{\partial \zeta }+v_{2}(\zeta ,{\Im })\frac{\partial v_{1}(\zeta ,{\Im })}{\partial \zeta }+v_{1}(\zeta ,{\Im })\frac{\partial v_{2}(\zeta ,{\Im })}{\partial \zeta }=0, \end{aligned} \end{aligned}$$

5 $$\begin{aligned} \begin{aligned}{}&D_{{\Im }}^{{\varrho }} v_{2}(\zeta , {\Im })-\frac{\partial ^2 v_{2}(\zeta ,{\Im })}{\partial \zeta ^2}-2v_{2}(\zeta ,{\Im })\frac{\partial v_{2}(\zeta ,{\Im })}{\partial \zeta }+v_{2}(\zeta ,{\Im })\frac{\partial v_{1}(\zeta ,{\Im })}{\partial \zeta }+v_{1}(\zeta ,{\Im })\frac{\partial v_{2}(\zeta ,{\Im })}{\partial \zeta }=0, \end{aligned} \end{aligned}$$

where 0 < p ≤ 1.

Having the following initial condations:

6 $$\begin{aligned} \begin{aligned} v_1(\zeta , 0)=M(\zeta ),\ v_2(\zeta , 0)=N(\zeta ), \end{aligned} \end{aligned}$$

The RPSM was established in 2013 by Omar Abu Arqub^57^. The RPSM is a semi-analytical approach that brings together Taylor’s series and the residual error function. Any kind of differential equation, linear or nonlinear, may be solved using the provided convergence series algorithms. The first use of RPSM in 2013 was in the domain of fuzzy DE resolution. A novel set of RPSM algorithms was developed by Arqub et al.^58^ for the efficient discovery of power series solutions to complex DEs. Arqub et al.^59^ also created a novel RPSM approach that solves non-linear fractional PDE. A novel RPSM approach for fractional KdV-burgers equations was developed by El-Ajou et al.^60^. It was suggested by Xu et al.^61,62^ that fractional power series may be used to solve second- and fourth-order Boussinesq DEs. Zhang et al.^63^ developed a successful numerical approach by merging RPSM with least square methods^64–66^.

The solution of the fractional-order differential equation (FODEs) was obtained by combining new methods developed during the research. First, convert the given equation to a space known as Aboodh transform^67,68^. The different equations being updated are then solved for from which different solutions are obtained. Lastly, by applying the inverse Aboodh transform to the second equation, the first equation is solved. Here, the Sumudu approximation is combined with homotopy perturbation methods. The new power series expansion strategy, which does not demand linearization, perturbation, and discretization is applied to solve the linear and nonlinear differential equations. When it comes to identification of coefficients, there is no complicated procedure like in RPSM where finding fractional derivatives takes many solution iterations. This method could be able to reach a close approximate solution by applying a fast convergence series of the suggested approach.

The most significant mathematical accomplishment of the twentieth century was Aboodh’s transform iterative approach (NITM) for fractional partial differential equations. Partial differential equations with fractional derivatives are notoriously hard to solve using standard methods because of their computational complexity and non-convergence. By reducing processing effort, enhancing accuracy, and continuously improving approximation solutions, our unique technology goes above these restrictions. Iterations tailored to fractional derivatives have improved the solutions to complex mathematical and physical problems^69–71^. Complicated PDEs systems have recently been developed, allowing for the study of physics, applied mathematics, and engineering problems that have traditionally been difficult to tackle.

## Foundations

### Definition 2.1

The function $v(\zeta , {{\Im }})$ is exponentially ordered and has piecewise continuity^72^. For $v(\zeta , {{\Im }})$, the Aboodh transform (AT) is defined as follows where ${\Im }\ge 0$:

$$\begin{aligned} A[v({\zeta }, {{\Im }})]=\Psi ({\zeta },\mu )=\frac{1}{\mu }\int _{0}^{\infty }v({\zeta }, {{\Im }})e^{-{{\Im }}\mu }d{{\Im }},\ \ {r}_{1}\le \mu \le {r}_{2}, \end{aligned}$$

The following is the formula for the Inverse Aboodh transform (IAT):

$$\begin{aligned} A^{-1}[\Psi ({\zeta },\mu )]=v({\zeta }, {{\Im }})=\frac{1}{2\pi i}\int _{u-i\infty }^{u+i\infty }\Psi ({\zeta }, {{\Im }})\mu e^{{{\Im }}\mu }d{{\Im }} \end{aligned}$$

where $${\zeta }=({\zeta }_{1},{\zeta }_{2},\ldots ,{\zeta }_{{\varrho }})$$ and $$p\in \mathbb {N}$$.

### Lemma 2.1

^73,74^ Take $v_{1}({\zeta }, {{\Im }})$ and $v_{2}({\zeta }, {{\Im }})$ as functions that are piecewise continuous over the interval $[0,\infty [$ and that have exponential order, correspondingly. The assumption being that

$\Psi _{1}({\zeta }, {{\Im }})=A[v_{1}({\zeta }, {{\Im }})]$ and $\Psi _{2}({\zeta }, {{\Im }})=A[v_{2}({\zeta }, {{\Im }})]$. We have constants $\varpi _{1},\varpi _{2}$. Therefore, the below statements hold true:

1. $$A[\varpi _{1}v_{1}({\zeta }, {{\Im }})+\varpi _{2}v_{2}({\zeta }, {{\Im }})]=\varpi _{1}\Psi _{1}({\zeta },\mu )+\varpi _{2}\Psi _{2}({\zeta }, {\mu })$$,
2. $$A^{-1}[\varpi _{1}\Psi _{1}({\zeta }, {{\Im }})+\varpi _{2}\Psi _{2}({\zeta }, {{\Im }})]=\varpi _{1}v_{1}({\zeta },\mu )+\varpi _{2}v_{2}({\zeta }, {\mu })$$,
3. $$A[J_{{{\Im }}}^{{{\varrho }}}v({\zeta }, {{\Im }})]=\frac{\Psi ({\zeta },\mu )}{\mu ^{{{\varrho }}}}$$,
4. $$A[D_{{{\Im }}}^{{{\varrho }}}v({\zeta }, {{\Im }})]=\mu ^{{{\varrho }}}\Psi ({\zeta },\mu )-\sum _{K=0}^{r-1}\frac{v^{K}({\zeta },0)}{\mu ^{K-{{\varrho }}+2}}, r-1<{{\varrho }}\le r,\ r\in \mathbb {N}$$.

### Definition 2.2

A function $v({\zeta }, {{\Im }})$ of order ${{\varrho }}$ may be defined, in the Caputo sense, as follows^75^:

$$\begin{aligned} D_{{{\Im }}}^{{{\varrho }}}v({\zeta }, {{\Im }})=J_{{{\Im }}}^{m-{{\varrho }}}v^{(m)}({\zeta }, {{\Im }}), \ r\ge 0, \ m-1<{{\varrho }}\le m, \end{aligned}$$

where ${\zeta }=({\zeta }_{1},{\zeta }_{2},\ldots , {\zeta }_{{\varrho }})\in \mathbb {R}^{{\varrho }}$ and $m,p\in R, J_{{{\Im }}}^{m-{{\varrho }}}$ is the R-L integral of $v({\zeta }, {{\Im }})$.

### Definition 2.3

^76^ The power series may be expressed as:

$$\begin{aligned} \sum _{r=0}^{\infty }\hbar _{r}({\zeta })({{\Im }}-{{\Im }}_{0})^{r{{\varrho }}}=\hbar _{0}({{\Im }}-{{\Im }}_{0})^{0}+\hbar _{1}({{\Im }}-{{\Im }}_{0})^{{{\varrho }}}+\hbar _{2}({{\Im }}-{{\Im }}_{0})^{2{{\varrho }}} +\cdots , \end{aligned}$$

${\zeta }=({\zeta }_{1},{\zeta }_{2},\ldots , {\zeta }_{{\varrho }})\in \mathbb {R}^{{\varrho }}$, where $p\in \mathbb {N}$. This series is recognised as an MFPS about ${{\Im }}_{0}$, where ${{\Im }}$ is a variable and the series coefficients are indicated by $\hbar _{r}({\zeta })$.

### Lemma 2.2

Assuming the function $v({\zeta }, {{\Im }})$ has an exponential order, the Aboodh transform is denoted as $A[v({\zeta }, {{\Im }})]=\Psi ({\zeta },\mu )$. Due to this

7 $$\begin{aligned} A[D_{{{\Im }}}^{r{{\varrho }}}v({\zeta }, {{\Im }})]=\mu ^{r{{\varrho }}}\Psi ({\zeta },\mu )-\sum _{j=0}^{r-1}\mu ^{{{\varrho }}(r-j)-2}D_{{{\Im }}}^{j{{\varrho }}}v({\zeta },0), 0<{{\varrho }}\le 1, \end{aligned}$$

where ${\zeta }=({\zeta }_{1},{\zeta }_{2},\ldots , {\zeta }_{{\varrho }})\in \mathbb {R}^{{\varrho }}$ and $p\in \mathbb {N}$ and $D_{{{\Im }}}^{r{{\varrho }}}=D_{{{\Im }}}^{{{\varrho }}}.D_{{{\Im }}}^{{{\varrho }}}.\cdots .D_{{{\Im }}}^{{{\varrho }}}(r-times)$.

### Proof

We will use the induction method to verify Eq. (2). The following result is obtained when $r=1$ is substituted into Eq. (2).

$$\begin{aligned} A[D_{{{\Im }}}^{2{{\varrho }}}v({\zeta }, {{\Im }})]=\mu ^{2{{\varrho }}}\Psi ({\zeta },\mu )-\mu ^{2{{\varrho }}-2}v({\zeta },0)-\mu ^{{{\varrho }}-2}D_{{{\Im }}}^{{{\varrho }}}v({\zeta },0). \end{aligned}$$

Lemma 2.1, specifically Part (4), proves that the equation is true for $r=1$. We get: by putting $r=2$ into Eq. (2).

8 $$\begin{aligned} A[D_{r}^{2{{\varrho }}}v({\zeta }, {{\Im }})]=\mu ^{2{{\varrho }}}\Psi ({\zeta },\mu )-\mu ^{2{{\varrho }}-2}v({\zeta },0)-\mu ^{{{\varrho }}-2}D_{{{\Im }}}^{{{\varrho }}}v({\zeta },0). \end{aligned}$$

Taking into consideration the L.H.S. of Eq. (8), the result is:

9 $$\begin{aligned} L.H.S=A[D_{{{\Im }}}^{2{{\varrho }}}v({\zeta }, {{\Im }})]. \end{aligned}$$

There is a specific way to write Eq. (9) as:

10 $$\begin{aligned} L.H.S=A[D_{{{\Im }}}^{{{\varrho }}}v({\zeta }, {{\Im }})]. \end{aligned}$$

Let

11 $$\begin{aligned} z({\zeta }, {{\Im }})=D_{{{\Im }}}^{{{\varrho }}}v({\zeta }, {{\Im }}). \end{aligned}$$

As a result, Eq. (10) transforms to:

12 $$\begin{aligned} L.H.S=A[D_{{{\Im }}}^{{{\varrho }}}z({\zeta }, {{\Im }})]. \end{aligned}$$

Supported by a fractional derivative of the Caputo type.

13 $$\begin{aligned} L.H.S=A[J^{1-{{\varrho }}}z^{'}({\zeta }, {{\Im }})]. \end{aligned}$$

The formula for the R-L integral of the Aboodh transform is determined by Eq. (13).

14 $$\begin{aligned} L.H.S=\frac{A[z^{'}({\zeta }, {{\Im }})]}{\mu ^{1-{{\varrho }}}}. \end{aligned}$$

By making use of the Aboodh transform’s differential property, Eq. (14) changed to:

15 $$\begin{aligned} L.H.S= \mu ^{{{\varrho }}}Z({\zeta },\mu )-\frac{z({\zeta },0)}{\mu ^{2-{{\varrho }}}}, \end{aligned}$$

We obtain: using Eq. (11) as a guide.

$$\begin{aligned} Z({\zeta },\mu )=\mu ^{{{\varrho }}}\Psi ({\zeta },\mu )-\frac{v({\zeta },0)}{\mu ^{2-{{\varrho }}}}, \end{aligned}$$

in the case when $A[z({\zeta }, {{\Im }})]=Z({\zeta },\mu )$. Equation (15) is therefore changed into

16 $$\begin{aligned} L.H.S=\mu ^{2{{\varrho }}}\Psi ({\zeta },\mu )-\frac{v({\zeta },0)}{\mu ^{2-2{{\varrho }}}}-\frac{D_{{{\Im }}}^{{{\varrho }}}v({\zeta },0)}{\mu ^{2-{{\varrho }}}}, \end{aligned}$$

There is compatibility between Eqs. (2) and (16). With the assumption that Eq. (2) is valid for $r=K$. Equation (2) may be revised by substituting $r=K$.

17 $$\begin{aligned} A[D_{{{\Im }}}^{K{{\varrho }}}v({\zeta }, {{\Im }})]=\mu ^{K{{\varrho }}}\Psi ({\zeta },\mu )-\sum _{j=0}^{K-1}\mu ^{{{\varrho }}(K-j)-2}D_{{{\Im }}}^{j{{\varrho }}}D_{{{\Im }}}^{j{{\varrho }}}v({\zeta },0),\ 0<{{\varrho }}\le 1. \end{aligned}$$

We shall show that Eq. (2) is true for $r=K+1$ below.

18 $$\begin{aligned} A[D_{{{\Im }}}^{(K+1){{\varrho }}}v({\zeta }, {{\Im }})]=\mu ^{(K+1){{\varrho }}}\Psi ({\zeta },\mu )-\sum _{j=0}^{K}\mu ^{{{\varrho }}((K+1)-j)-2}D_{{{\Im }}}^{j{{\varrho }}}v({\zeta },0). \end{aligned}$$

In Eq. (18), on the left-hand side, we get

19 $$\begin{aligned} L.H.S= A[D_{{{\Im }}}^{K{{\varrho }}}(D_{{{\Im }}}^{K{{\varrho }}})]. \end{aligned}$$

Let

$$\begin{aligned} D_{{{\Im }}}^{K{{\varrho }}}=g({\zeta }, {{\Im }}). \end{aligned}$$

Equation (19) gives

20 $$\begin{aligned} L.H.S= A[D_{{{\Im }}}^{{{\varrho }}}g({\zeta }, {{\Im }})]. \end{aligned}$$

One possible way to express Eq. (20) is by combining the Caputo fractional derivative with the R-L integral.

21 $$\begin{aligned} L.H.S=\mu ^{{{\varrho }}}A[D_{{{\Im }}}^{K{{\varrho }}}v({\zeta }, {{\Im }})]-\frac{g({\zeta },0)}{\mu ^{2-{{\varrho }}}}. \end{aligned}$$

After Eq. (17) is applied, Eq. (21) is changed into

22 $$\begin{aligned} L.H.S=\mu ^{r{{\varrho }}}\Psi ({\zeta },\mu )-\sum _{j=0}^{r-1}\mu ^{{{\varrho }}(r-j)-2}D_{{{\Im }}}^{j{{\varrho }}}v({\zeta },0), \end{aligned}$$

The following outcome is obtained by using Eq. (22).

$$\begin{aligned} L.H.S= A[D_{{{\Im }}}^{r{{\varrho }}}v({\zeta },0)]. \end{aligned}$$

This proves that Eq. (2) is valid for $r=K+1$. Therefore, for all positive integers, the mathematical induction approach was used to prove that Eq. (2) is true. $\square$

The following lemma illustrates a new form of the ARPSM multiple fractional Taylor’s formula.

### Lemma 2.3

Let the exponentially ordered function $v({\zeta }, {{\Im }})$ be defined. The set $A[v({\zeta }, {{\Im }})]= \Psi ({\zeta },\mu )$ is the Aboodh transform of the multiple fractional Taylor’s series of $v({\zeta }, {{\Im }})$.

23 $$\begin{aligned} \Psi ({\zeta },\mu )=\sum _{r=0}^{\infty }\frac{\hbar _{r}({\zeta })}{\mu ^{r{{\varrho }}+2}}, \mu >0, \end{aligned}$$

where, $${\zeta }=(\zeta _{1},{\zeta }_{2},\ldots ,{\zeta }_{{\varrho }})\in \mathbb {R}^{{\varrho }}, \ p\in \mathbb {N}$$.

### Proof

The results of the fractional order analysis of Taylor’s series are as follows:

24 $$\begin{aligned} v({\zeta }, {{\Im }})=\hbar _{0}({\zeta })+\hbar _{1}({\zeta })\frac{{{\Im }}^{{{\varrho }}}}{\Gamma [{{\varrho }}+1]}++\hbar _{2}({\zeta })\frac{{{\Im }}^{2{{\varrho }}}}{\Gamma [2{{\varrho }}+1]}+\cdots . \end{aligned}$$

This equivalence may be obtained by using the Aboodh transform on Eq. (24).

$$\begin{aligned} A\left[ v({\zeta }, {{\Im }})\right] =A\left[ \hbar _{0}({\zeta })\right] +A\left[ \hbar _{1}({\zeta })\frac{{{\Im }}^{{{\varrho }}}}{\Gamma [{{\varrho }}+1]}\right] +A\left[ \hbar _{1}({\zeta }) \frac{{{\Im }}^{2{{\varrho }}}}{\Gamma [2{{\varrho }}+1]}\right] +\cdots \end{aligned}$$

These characteristics of the Aboodh transform are used to get

$$\begin{aligned} A\left[ v({\zeta }, {{\Im }})\right] =\hbar _{0}({\zeta })\frac{1}{\mu ^{2}}+\hbar _{1}({\zeta })\frac{\Gamma [{{\varrho }}+1]}{\Gamma [{{\varrho }}+1]}\frac{1}{\mu ^{{{\varrho }}+2}}+\hbar _{2}({\zeta }) \frac{\Gamma [2{{\varrho }}+1]}{\Gamma [2{{\varrho }}+1]}\frac{1}{\mu ^{2{{\varrho }}+2}}\cdots \end{aligned}$$

We get 23, a new set of Taylor’s in the Aboodh transformation, because of this. $\square$

### Lemma 2.4

Based on the new form of Taylor’s series 23, we may assume that the MFPS notation of the function $A[v({\zeta }, {{\Im }})] = \Psi ({\zeta }, \mu )$ exists.

25 $$\begin{aligned} \hbar _{0}({\zeta })=\lim _{\mu \rightarrow \infty }\mu ^2\Psi ({\zeta },\mu )=v({\zeta },0). \end{aligned}$$

### Proof

Taylor’s series has been revised and serves as the basis for this previously.

26 $$\begin{aligned} \hbar _{0}({\zeta })=\mu ^2\Psi ({\zeta },\mu )-\frac{\hbar _{1}({\zeta })}{\mu ^{{{\varrho }}}}-\frac{\hbar _{2}({\zeta })}{\mu ^{2{{\varrho }}}}-\cdots \end{aligned}$$

By applying $\lim _{\mu \rightarrow \infty }$ to Eq. (25) and doing certain computations, the intended result, represented by 26, is achieved. $\square$

### Theorem 2.5

The MFPS representations of the functions $v({\zeta }, {{\Im }})$ and $\Psi ({\zeta },\mu )$ are as follows:

$$\begin{aligned} \Psi ({\zeta },\mu )=\sum _{0}^{\infty }\frac{\hbar _{r}({\zeta })}{\mu ^{r{{\varrho }}+2}}, \ \mu >0, \end{aligned}$$

where ${\zeta }=({\zeta }_{1},{\zeta }_{2},\ldots ,{\zeta }_{{\varrho }})\in \mathbb {R}^p$ and $p\in \mathbb {N}$. Then we have

$$\begin{aligned} \hbar _{r}({\zeta })=D_{r}^{r{{\varrho }}}v({\zeta },0), \end{aligned}$$

where, $$D_{{{\Im }}}^{r{{\varrho }}}=D_{{{\Im }}}^{{{\varrho }}}.D_{{{\Im }}}^{{{\varrho }}}.\cdots .D_{{{\Im }}}^{{{\varrho }}}(r-times)$$.

### Proof

With the modified Taylor’s series form, we get

27 $$\begin{aligned} \hbar _{1}({\zeta })=\mu ^{{{\varrho }}+2}\Psi ({\zeta },\mu )-\mu ^{{{\varrho }}}\hbar _{0}({\zeta })-\frac{\hbar _{2}({\zeta })}{\mu ^{{{\varrho }}}}-\frac{\hbar _{3}({\zeta })}{\mu ^{2{{\varrho }}}}- \cdots \end{aligned}$$

One may solve Eq. (27) for $\lim _{\mu \rightarrow \infty }$ to get

$$\begin{aligned} \hbar _{1}({\zeta })=\lim _{\mu \rightarrow \infty }(\mu ^{{{\varrho }}+2}\Psi ({\zeta },\mu )-\mu ^{{{\varrho }}}\hbar _{0}({\zeta }))-\lim _{\mu \rightarrow \infty }\frac{\hbar _{2}({\zeta })}{\mu ^{{{\varrho }}}} -\lim _{\mu \rightarrow \infty }\frac{\hbar _{3}({\zeta })}{\mu ^{2{{\varrho }}}}- \cdots \end{aligned}$$

The following equality results from taking the limit:

28 $$\begin{aligned} \hbar _{1}({\zeta })=\lim _{\mu \rightarrow \infty }(\mu ^{{{\varrho }}+2}\Psi ({\zeta },\mu )-\mu ^{{{\varrho }}}\hbar _{0}({\zeta })). \end{aligned}$$

When Lemma 2.2 is combined with Eq. (28), the following result is obtained:

29 $$\begin{aligned} \hbar _{1}({\zeta })=\lim _{\mu \rightarrow \infty }(\mu ^2A[D_{{{\Im }}}^{{{\varrho }}}v({\zeta }, {{\Im }})](\mu )). \end{aligned}$$

Additionally, using Lemma 2.3 in conjunction with Eq. (29) yields the following result:

$$\begin{aligned} \hbar _{1}({\zeta })=D_{{{\Im }}}^{{{\varrho }}}v({\zeta },0). \end{aligned}$$

With $\mu \rightarrow \infty$ and the modified Taylor’s series, we get

$$\begin{aligned} \hbar _{2}({\zeta })=\mu ^{2{{\varrho }}+2}\Psi ({\zeta },\mu )-\mu ^{2{{\varrho }}}\hbar _{0}({\zeta })-\mu ^{{{\varrho }}}\hbar _{1}({\zeta })-\frac{\hbar _{3}({\zeta })}{\mu ^{{{\varrho }}}}- \cdots \end{aligned}$$

Lemma 2.3 is used to get

30 $$\begin{aligned} \hbar _2({\zeta })=\lim _{\mu \rightarrow \infty }\mu ^2(\mu ^{2{{\varrho }}}\Psi ({\zeta },\mu )-\mu ^{2{{\varrho }}-2}\hbar _{0}({\zeta })-\mu ^{{{\varrho }}-2}\hbar _{1}({\zeta })). \end{aligned}$$

Equation (30) is transformed using Lemmas 2.2 and 2.4 once again.

$$\begin{aligned} \hbar _{2}({\zeta })=D_{{{\Im }}}^{2{{\varrho }}}v({\zeta },0). \end{aligned}$$

Applying the same process to modified Taylor’s series yields

$$\begin{aligned} \hbar _{3}({\zeta })=\lim _{\mu \rightarrow \infty }\mu ^2(A[D_{{{\Im }}}^{2{{\varrho }}}v({\zeta },{{\varrho }})](\mu )). \end{aligned}$$

The final equation is obtained by using Lemma 2.4.

$$\begin{aligned} \hbar _{3}({\zeta })=D_{{{\Im }}}^{3{{\varrho }}}v({\zeta },0). \end{aligned}$$

In general we get

$$\begin{aligned} \hbar _{r}({\zeta })=D_{{{\Im }}}^{r{{\varrho }}}v({\zeta },0). \end{aligned}$$

That concludes the proof. $\square$

We prove the criteria for the modified Taylor formula to converge, both necessary and sufficient, in the following theorem.

### Theorem 2.6

Proposed in Lemma 2.3 is a modified version of Multiple Fractional Taylor’s formula, denoted as $A [v({\zeta }, {{\Im }})]=\Psi ({\zeta }, \mu )$. For $(0 \ \mu \le s)$ with $0 \ {{\varrho }} \le 1$, the updated multiple fractional Taylor’s formula is consistent with the following inequality if $|\mu ^{a}A[D_{{{\Im }}}^{(K+1){{\varrho }}}v({\zeta }, {{\Im }})]|\le T$.

$$\begin{aligned} |R_{K}({\zeta },\mu )|\le \frac{T}{\mu ^{(K=1){{\varrho }}+2}}, \ 0<\mu \le s. \end{aligned}$$

### Proof

The following assumptions are used to begin the proof: $A[D_{{{\Im }}}^{r{{\varrho }}}v({\zeta }, {{\Im }})]$ is defined for $0 < \mu \le s$, where $r = 0, 1, 2, \ldots ,K + 1$. Consider $|\mu ^{2}A[D_{{{\Im }}^{K+1}}v({\zeta }, \tau )]| \le T$ holds for $0 < \mu \le s$ given the specified conditions. Take a look at the revised version of the relationship that came out of Taylor’s series:

31 $$\begin{aligned} R_K({\zeta },\mu )=\Psi ({\zeta },\mu )-\sum _{r=0}^K\frac{\hbar _r({\zeta })}{\mu ^{r{{\varrho }}+2}}. \end{aligned}$$

Applying Theorem 2.5 changes Eq. (31) to:

32 $$\begin{aligned} R_K({\zeta },\mu )=\Psi ({\zeta },\mu )-\sum _{r=0}^{K}\frac{D_{{{\Im }}}^{r{{\varrho }}}v({\zeta },0)}{\mu ^{r{{\varrho }}+2}}. \end{aligned}$$

$\mu ^{(K+1)a+2}$ is multiplied on both side of (32), we have

33 $$\begin{aligned} \mu ^{(K+1){{\varrho }}+2}R_K({\zeta },\mu )=\mu ^{2}(\mu ^{(K+1){{\varrho }}}\Psi ({\zeta },\mu )-\sum _{r=0}^{K}\mu ^{(K+1-r){{\varrho }}-2}D_{{{\Im }}}^{r{{\varrho }}}v({\zeta },0)). \end{aligned}$$

Using Lemma 2.2 in conjunction with Eq. (33) yields:

34 $$\begin{aligned} \mu ^{(K+1){{\varrho }}+2}R_K({\zeta },\mu )=\mu ^{2}A[D_{{{\Im }}}^{(K+1){{\varrho }}}v({\zeta }, {{\Im }})]. \end{aligned}$$

Equation (34), when taken as an absolute, yields:

35 $$\begin{aligned} |\mu ^{(K+1){{\varrho }}+2}R_K({\zeta },\mu )|=|\mu ^{2}A[D_{{{\Im }}}^{(K+1){{\varrho }}}v({\zeta }, {{\Im }})]|. \end{aligned}$$

Applying the criteria given in Eq. (35) results in the following outcome, thus:

36 $$\begin{aligned} \frac{-T}{\mu ^{(K+1){{\varrho }}+2}}\le R_K({\zeta },\mu )\le \frac{T}{\mu ^{(K+1){{\varrho }}+2}}. \end{aligned}$$

The desired outcome is produced by Eq. (36).

$$\begin{aligned} |R_K({\zeta },\mu )|\le \frac{T}{\mu ^{(K+1){{\varrho }}+2}}. \end{aligned}$$

This results in the new series having a defined convergence condition. $\square$

## Detailed description of the proposed approaches

### Using the ARPSM method to solve time-fractional PDEs

We address our general model by presenting the guiding principles of ARPSM.

*Step 1.* Construct a generalized form of the equation.

37 $$\begin{aligned} D_{{{\Im }}}^{q{{\varrho }}}v({\zeta }, {{\Im }})+\vartheta ({\zeta })N(v)-\zeta ({\zeta },v)=0, \end{aligned}$$

*Step 2.* The following is derived by transforming both sides of Eq. (37) using the Aboodh transform:

38 $$\begin{aligned} A[D_{{{\Im }}}^{q{{\varrho }}}v({\zeta }, {{\Im }})+\vartheta ({\zeta })N(v)-\zeta ({\zeta },v)]=0, \end{aligned}$$

Applying Lemma 2.3, we can modify Eq. (38).

39 $$\begin{aligned} \Psi ({\zeta },{s})=\sum _{j=0}^{q-1}\frac{D_{{{\Im }}}^{j}v({\zeta },0)}{{s}^{q{{\varrho }}+2}}-\frac{\vartheta ({\zeta })Y({s})}{{s}^{q{{\varrho }}}}+\frac{F({\zeta },{s})}{{s}^{q{{\varrho }}}}, \end{aligned}$$

where, $A[\zeta ({\zeta },v)]=F({\zeta },{s}),A[N(v)]=Y({s})$. *Step 3.* This form must be considered in order to get the

solution to Eq. (39).

$$\begin{aligned} \Psi ({\zeta }, {s}) =\sum _{r=0}^{\infty }\frac{\hbar _r({\zeta })}{{s}^{r{{\varrho }}+2}}, \ {s} > 0, \end{aligned}$$

*Step 4.* Continue by following these steps:

$$\begin{aligned} \hbar _0({\zeta })= \lim _{{s}\rightarrow \infty }{s}^2 \Psi ({\zeta }, {s}) = v({\zeta }, 0), \end{aligned}$$

Using the results from Theorem 2.6, we may conclude as follows.

$$\begin{aligned}{}& \hbar _{1}({\zeta }) = D_{{{\Im }}}^{{{\varrho }}}v({\zeta }, 0), \\& \hbar _{2}({\zeta }) = D_{{{\Im }}}^{2{{\varrho }}}v({\zeta }, 0), \\ & \quad\quad\quad\quad \vdots \\& \hbar _{w}({\zeta }) = D_{{{\Im }}}^{w{{\varrho }}}v({\zeta }, 0), \end{aligned}$$

*Step 5.* Follow these procedures to get the $\Psi ({\zeta }, {s})$ as the $K^{th}$-truncated series:

$$\begin{gathered} \Psi _{K} (\zeta ,s) = \sum\limits_{{r = 0}}^{K} {\frac{{\hbar _{r} (\zeta )}}{{s^{{r\rangle + 2}} }}} ,\;s > 0, \hfill \\ \Psi _{K} (\zeta ,s) = \frac{{\hbar _{0} (\zeta )}}{{s^{2} }} + \frac{{\hbar _{1} (\zeta )}}{{s^{{\rangle + 2}} }} + \cdots + \frac{{\hbar _{w} (\zeta )}}{{s^{{w\rangle + 2}} }} + \sum\limits_{{r = w + 1}}^{K} {\frac{{\hbar _{r} (\zeta )}}{{s^{{r\rangle + 2}} }}} , \hfill \\ \end{gathered}$$

*Step 6.* We need to evaluate the Aboodh residual function (ARF) of Eq. (39) separately from the $K^{th}$-truncated Aboodh residual function so that we may get:

$$\begin{aligned} ARes({\zeta }, {s}) = \Psi ({\zeta },{s})-\sum _{j=0}^{q-1}\frac{D_{{{\Im }}}^{j}v({\zeta },0)}{{s}^{j{{\varrho }}+2}}+\frac{\vartheta ({\zeta })Y({s})}{{s}^{j{{\varrho }}}}-\frac{F({\zeta },{s})}{{s}^{j{{\varrho }}}}, \end{aligned}$$

and

40 $$\begin{aligned} ARes_{K}({\zeta }, {s}) =\Psi _{K}({\zeta },{s})-\sum _{j=0}^{q-1}\frac{D_{{{\Im }}}^{j}v({\zeta },0)}{{s}^{j{{\varrho }}+2}}+\frac{\vartheta ({\zeta })Y({s})}{{s}^{j{{\varrho }}}}-\frac{F({\zeta },{s})}{{s}^{j{{\varrho }}}}. \end{aligned}$$

*Step 7.* Subsituate the expansion form of $\Psi _{K}({\zeta }, {s})$ into Eq. (40).

41 $$\begin{gathered} ARes_{K} (\zeta ,s) = \left( {\frac{{\hbar _{0} (\zeta )}}{{s^{2} }} + \frac{{\hbar _{1} (\zeta )}}{{s^{{{\varrho } + 2}} }} + \cdots + \frac{{\hbar _{w} (\zeta )}}{{s^{{w{\varrho } + 2}} }} + \sum\limits_{{r = w + 1}}^{K} {\frac{{\hbar _{r} (\zeta )}}{{s^{{r{\varrho } + 2}} }}} } \right) \hfill \\ \quad \quad \quad \quad \quad \quad\quad\; - \sum\limits_{{j = 0}}^{{q - 1}} {\frac{{D_{\Im }^{j} v(\zeta ,0)}}{{s^{{j{\varrho } + 2}} }}} + \frac{{\vartheta (\zeta )Y(s)}}{{s^{{j{\varrho }}} }} - \frac{{F(\zeta ,s)}}{{s^{{j{\varrho }}} }}. \hfill \\ \end{gathered}$$

*Step 8.* In Eq. (41), multiply ${s}^{K{{\varrho }}+2}$ on both sides.

42 $$\begin{gathered} s^{{K{\varrho } + 2}} ARes_{K} (\zeta ,s) = s^{{K{\varrho } + 2}} \left( {\frac{{\hbar _{0} (\zeta )}}{{s^{2} }} + \frac{{\hbar _{1} (\zeta )}}{{s^{{{\varrho } + 2}} }} + \cdots + \frac{{\hbar _{w} (\zeta )}}{{s^{{w{\varrho } + 2}} }} + \sum\limits_{{r = w + 1}}^{K} {\frac{{\hbar _{r} (\zeta )}}{{s^{{r{\varrho } + 2}} }}} } \right. \hfill \\ \left. {\quad \quad \quad \quad \quad \quad \quad \quad \quad \quad - \sum\limits_{{j = 0}}^{{q - 1}} {\frac{{D_{\Im }^{j} v(\zeta ,0)}}{{s^{{j{\varrho } + 2}} }}} + \frac{{\vartheta (\zeta )Y(s)}}{{s^{{j{\varrho }}} }} - \frac{{F(\zeta ,s)}}{{s^{{j{\varrho }}} }}} \right). \hfill \\ \end{gathered}$$

*Step 9.* This evaluation of Eq. (42) is being done with regard to $\lim _{{s}\rightarrow \infty }$ on both sides.

$$\begin{gathered} \mathop {\lim }\limits_{{s \to \infty }} s^{{K{\varrho } + 2}} ARes_{K} (\zeta ,s) = \mathop {\lim }\limits_{{s \to \infty }} s^{{K{\varrho } + 2}} \left( {\frac{{\hbar _{0} (\zeta )}}{{s^{2} }} + \frac{{\hbar _{1} (\zeta )}}{{s^{{{\varrho } + 2}} }} + \cdots + \frac{{\hbar _{w} (\zeta )}}{{s^{{w{\varrho } + 2}} }} + \sum\limits_{{r = w + 1}}^{K} {\frac{{\hbar _{r} (\zeta )}}{{s^{{r{\varrho } + 2}} }}} } \right. \hfill \\ \left. {\quad \quad \quad \quad \quad \quad \quad \quad \quad \quad \quad\quad - \sum\limits_{{j = 0}}^{{q - 1}} {\frac{{D_{\Im }^{j} v(\zeta ,0)}}{{s^{{j{\varrho } + 2}} }}} + \frac{{\vartheta (\zeta )Y(s)}}{{s^{{j{\varrho }}} }} - \frac{{F(\zeta ,s)}}{{s^{{j{\varrho }}} }}} \right). \hfill \\ \end{gathered}$$

*Step 10.* To get $\hbar _K({\zeta })$, the following equation must be solved.

$$\begin{aligned} \lim _{{s}\rightarrow \infty }({s}^{K{{\varrho }}+2}ARes_{K}({\zeta }, {s}))=0, \end{aligned}$$

where $$K=w+1,w+2,\ldots .$$

*Step 11.* By putting the values of $\hbar _K({\zeta })$ into the *K*-truncated series of $\Psi ({\zeta }, {s})$, we can get the *K*-approximate solution for Eq. (39).

*Step 12.* The *K*-approximate solution $v_K({\zeta }, {{\Im }})$ might be obtained by applying an inverse Aboodh transform to $\Psi _K({\zeta }, {s})$.

###  Problem 1

Take the homogeneous form of the mKdV equation into consideration.

43 $$\begin{aligned} \begin{aligned}{}&D_{{\Im }}^{{\varrho }} v_{1}(\zeta , {\Im })+6v_{1}^{2}(\zeta ,{\Im })\frac{\partial v_{1}(\zeta ,{\Im })}{\partial \zeta }+\frac{\partial ^{3} v_{1}(\zeta ,{\Im })}{\partial \zeta ^{3}}=0, \end{aligned} \end{aligned}$$

Having the following IC’s:

44 $$\begin{aligned} \begin{aligned} v_{1}(\zeta , 0)=\sqrt{c}\ \text {sech}\left( k+\sqrt{c} \zeta \right) , \end{aligned} \end{aligned}$$

and exact solution

45 $$\begin{aligned} \begin{aligned} v_{1}(\zeta , {\Im })=\sqrt{c}\ \text {sech}\left( k+\sqrt{c} (\zeta -c{\Im })\right) . \end{aligned} \end{aligned}$$

By applying Eq. (44) and the Aboodh Transform (AT) on Eq. (43), we are able to derive:

46 $$\begin{aligned} \begin{aligned}{}&v_{1}(\zeta , s)-\frac{\sqrt{c}\ \text {sech}\left( k+\sqrt{c} \zeta \right) }{s^2}+\frac{6}{s^p}\mathcal {A}_{{\Im }}\Bigg [\mathcal {A}_{{\Im }}^{-1}v_{1}(\zeta ,{\Im })\frac{\partial \mathcal {A}_{{\Im }}^{-1} v_{1}(\zeta ,{\Im })}{\partial \zeta }\Bigg ]+\frac{1}{s^p}\mathcal {A}_{{\Im }}\Bigg [\frac{\partial ^{3}\mathcal {A}_{{\Im }}^{-1} v_{1}(\zeta ,{\Im })}{\partial \zeta ^{3}}\Bigg ]=0. \end{aligned} \end{aligned}$$

The $k^{th}$ truncated term series are

47 $$\begin{aligned} \begin{aligned} v_{1}({\zeta },s)=\frac{\sqrt{c}\ \text {sech}\left( k+\sqrt{c} \zeta \right) }{s^2}+\sum _{r=1}^{k}\frac{f_{r}({\zeta },s)}{s^{rp+1}}, \ \ r=1,2,3,4\ldots . \end{aligned} \end{aligned}$$

Aboodh residual functions (ARFs) are

48 $$\begin{aligned} \begin{aligned}{}&\mathcal {A}_{{{\Im }}}Res({\zeta },s)=v_{1}(\zeta , t)-\frac{\sqrt{c}\ \text {sech}\left( k+\sqrt{c} \zeta \right) }{s^2}+\frac{6}{s^p}\mathcal {A}_{{\Im }}\Bigg [\mathcal {A}_{{\Im }}^{-1}v_{1}(\zeta ,{\Im })\frac{\partial \mathcal {A}_{{\Im }}^{-1} v_{1}(\zeta ,{\Im })}{\partial \zeta }\Bigg ]\\&\quad \quad \quad \quad \quad \quad \;\;+\frac{1}{s^p}\mathcal {A}_{{\Im }}\Bigg [\frac{\partial ^{3}\mathcal {A}_{{\Im }}^{-1} v_{1}(\zeta ,{\Im })}{\partial \zeta ^{3}}\Bigg ]=0\\ \end{aligned} \end{aligned}$$

and the *k*th-LRFs as:

49 $$\begin{aligned} \begin{aligned}{}&\mathcal {A}_{{{\Im }}}Res_{k}({\zeta },s)={v_{1}}_{k}(\zeta , t)-\frac{\sqrt{c}\ \text {sech}\left( k+\sqrt{c} \zeta \right) }{s^2}+\frac{6}{s^p}\mathcal {A}_{{\Im }}\Bigg [\mathcal {A}_{{\Im }}^{-1}{v_{1}}_{k}(\zeta ,{\Im })\frac{\partial \mathcal {A}_{{\Im }}^{-1} {v_{1}}_{k}(\zeta ,{\Im })}{\partial \zeta }\Bigg ]\\&\quad \quad \quad \quad \quad \quad \quad +\frac{1}{s^p}\mathcal {A}_{{\Im }}\Bigg [\frac{\partial ^{3}\mathcal {A}_{{\Im }}^{-1} {v_{1}}_{k}(\zeta ,{\Im })}{\partial \zeta ^{3}}\Bigg ]=0\\ \end{aligned} \end{aligned}$$

To calculate $f_{r}({\zeta },s)$, do the following procedures: The $r^{th}$-truncated series from Eq. (47) should be substituted into the $r^{th}$-Aboodh residual function depicted in Eq. (49), and the resultant equations should be multiplied by $s^{r{{\varrho }}+1}$. The relation $\lim _{s\rightarrow \infty }(s^{r{{\varrho }}+1} {A}{{{\Im }}}Res{v_{1},r}({\zeta },s))=0$ is then solved iteratively.in the case of $r = 1, 2, 3, \ldots$. The following are the first few terms:

50 $$\begin{aligned} \begin{aligned}{}&f_{1}({\zeta },s)=\frac{1}{2} c^2 \left( \cosh \left( 2 \left( \sqrt{c} \zeta +k\right) \right) -11\right) \tanh \left( \sqrt{c} \zeta +k\right) \text {sech}^3\left( \sqrt{c} \zeta +k\right) , \end{aligned} \end{aligned}$$

51 $$\begin{aligned} \begin{aligned}{}&f_{2}(\zeta ,s)=\frac{1}{32} c^{7/2} \Big (10543 \cosh \Big (2 \Big (\sqrt{c} \zeta +k\Big )\Big )-722 \cosh \Big (4 \Big (\sqrt{c} \zeta +k\Big )\Big )\\&\quad \quad \quad \quad \quad +\cosh \Big (6 \Big (\sqrt{c} \zeta +k\Big )\Big )-11774\Big ) \text {sech}^7\Big (\sqrt{c} \zeta +k\Big ). \end{aligned} \end{aligned}$$

and so on.

Putting the values of $f_{r}({\zeta },s)$, $r=1,2,3,\ldots ,$ in Eq. (47), we get

52 $$\begin{aligned} \begin{aligned}{}&v_{1}(\zeta ,s)=\Big (c^{7/2} \Big (10543 \cosh \Big (2 \Big (\sqrt{c} x+k\Big )\Big )-722 \cosh \Big (4 \Big (\sqrt{c} x+k\Big )\Big )\\&\quad \quad \quad \quad \quad +\cosh \Big (6 \Big (\sqrt{c} x+k\Big )\Big )-11774\Big ) \text {sech}^7\Big (\sqrt{c} x+k\Big )\Big )/\big (32 s^{2 p+1}\big )\\&\quad \quad \quad \quad \quad +\frac{c^2 \Big (\cosh \Big (2 \Big (\sqrt{c} x+k\Big )\Big )-11\Big ) \tanh \Big (\sqrt{c} x+k\Big ) \text {sech}^3\Big (\sqrt{c} x+k\Big )}{2 s^{p+1}}+\frac{\sqrt{c}\ \text {sech}\Big (\sqrt{c} x+k\Big )}{s}+\cdots .\\ \end{aligned} \end{aligned}$$

Applying the inverse transform of Aboodh yields

53 $$\begin{aligned} \begin{aligned}{}&v_{1}(\zeta ,{\Im })={\Im }^{2p}\Big (c^{7/2} \Big (10543 \cosh \Big (2 \Big (\sqrt{c} x+k\Big )\Big )-722 \cosh \Big (4 \Big (\sqrt{c} x+k\Big )\Big )\\&\quad \quad \quad \quad \quad +\cosh \Big (6 \Big (\sqrt{c} x+k\Big )\Big )-11774\Big ) \text {sech}^7\Big (\sqrt{c} x+k\Big )\Big )/(32 \Gamma (2 p+1))\\ {}&\quad \quad \quad \quad \quad +\frac{c^2{\Im }^{{\varrho }} \Big (\cosh \Big (2 \Big (\sqrt{c} x+k\Big )\Big )-11\Big ) \tanh \Big (\sqrt{c} x+k\Big ) \text {sech}^3\Big (\sqrt{c} x+k\Big )}{2 \Gamma (p+1)}+\sqrt{c}\ \text {sech}\Big (\sqrt{c} x+k\Big )+\cdots . \end{aligned} \end{aligned}$$

**Table 1:** Analysis of the ARPSM solution for various fractional order of example 1 for $v_{1}(\zeta ,{\Im })$ for ${\Im }=0.1$.

$\zeta$	$A ARPSM_{{P = 0.6}}$	$ARPSM_{p=0.8}$	$ARPSM_{P=1.0}$	Exact	Error of $p=0.6$	Error of $p=0.8$	Error of $p=1.0$
0.1	0.223099	0.223387	0.223529	0.223549	0.000449467	0.000161203	0.0000195862
0.2	0.222806	0.223146	0.223343	0.223379	0.000572584	0.0002325	0.0000362061
0.3	0.222407	0.222796	0.223045	0.223098	0.00069067	0.000301982	0.0000526159
0.4	0.221903	0.222337	0.222638	0.222707	0.000803136	0.00036927	0.0000687278
0.5	0.221296	0.221772	0.222121	0.222206	0.000909476	0.000434018	0.0000844573
0.6	0.220587	0.2211	0.221497	0.221596	0.00100927	0.000495911	0.0000997242
0.7	0.219778	0.220325	0.220766	0.22088	0.00110218	0.000554669	0.000114453
0.8	0.218871	0.219449	0.21993	0.220059	0.00118795	0.00061005	0.000128575
0.9	0.217868	0.218473	0.218993	0.219135	0.00126642	0.000661848	0.000142027
1.	0.216772	0.2174	0.217955	0.21811	0.0013375	0.000709894	0.000154751

**Figure 1:**
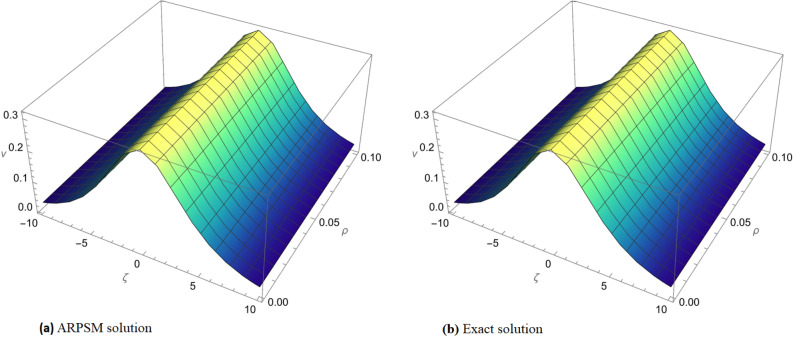
Comparison of the exact solution and approximate solution of $v_{1}(\zeta ,{\Im })$ for ${\Im }=0.1$.

**Figure 2:**
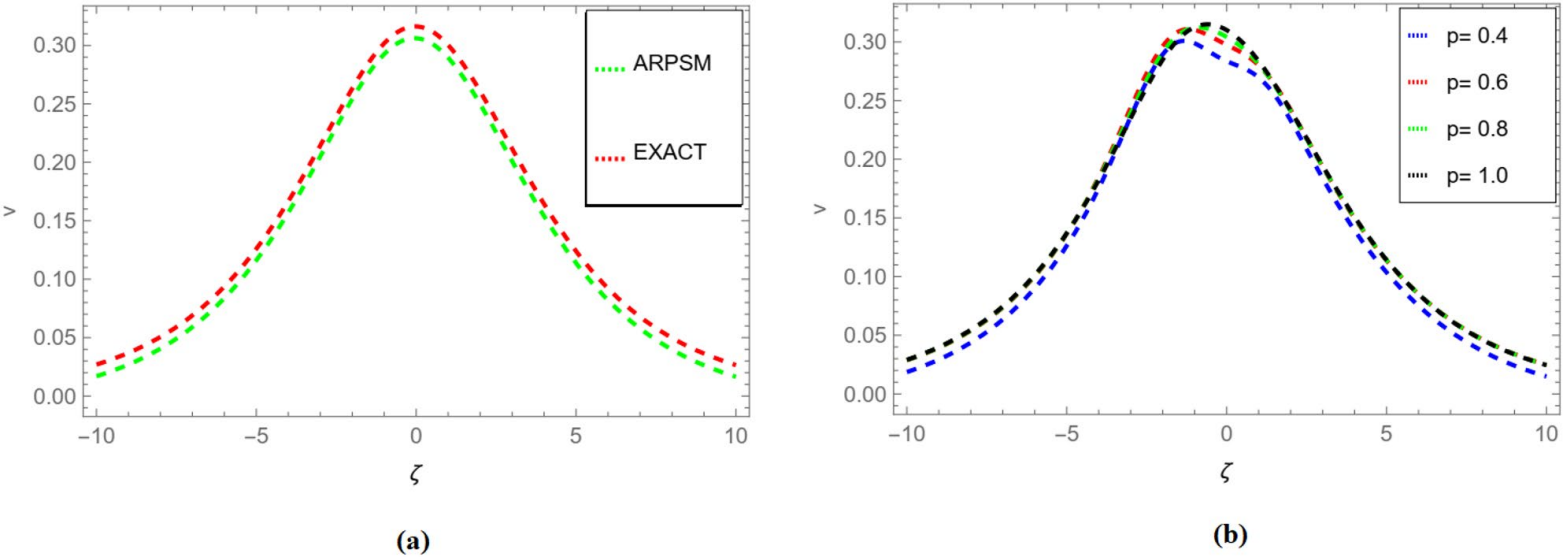
(**a**) Comparison of the exact solution and approximate solution. (**b**) Fractional order comparison of $v_{1}(\zeta ,{\Im })$ at ${\Im }=0.1$.

###  Problem 2

Analyze the following coupled system of homogeneous Burger’s equations:

54 $$\begin{aligned} \begin{aligned}{}&D_{{\Im }}^{{\varrho }} v_{1}(\zeta , {\Im })-\frac{\partial ^2 v_{1}(\zeta ,{\Im })}{\partial \zeta ^2}-2v_{1}(\zeta ,{\Im })\frac{\partial v_{1}(\zeta ,{\Im })}{\partial \zeta }+v_{2}(\zeta ,{\Im })\frac{\partial v_{1}(\zeta ,{\Im })}{\partial \zeta }+v_{1}(\zeta ,{\Im })\frac{\partial v_{2}(\zeta ,{\Im })}{\partial \zeta }=0, \end{aligned} \end{aligned}$$

55 $$\begin{aligned} \begin{aligned}{}&D_{{\Im }}^{{\varrho }} v_{2}(\zeta , {\Im })-\frac{\partial ^2 v_{2}(\zeta ,{\Im })}{\partial \zeta ^2}-2v_{2}(\zeta ,{\Im })\frac{\partial v_{2}(\zeta ,{\Im })}{\partial \zeta }+v_{2}(\zeta ,{\Im })\frac{\partial v_{1}(\zeta ,{\Im })}{\partial \zeta }+v_{1}(\zeta ,{\Im })\frac{\partial v_{2}(\zeta ,{\Im })}{\partial \zeta }=0, \ \\ {}&\text{ where } \ \ 0<p \le 1 \end{aligned} \end{aligned}$$

Having IC’s:

56 $$\begin{aligned} \begin{aligned} v_1(\zeta , 0)=sin(\zeta ), \end{aligned} \end{aligned}$$

57 $$\begin{aligned} \begin{aligned} v_2(\zeta , 0)=sin(\zeta ), \end{aligned} \end{aligned}$$

and exact solution

58 $$\begin{aligned} \begin{aligned} v_1(\zeta , {\Im })=e^{-{\Im }}\sin (\zeta ), \end{aligned} \end{aligned}$$

59 $$\begin{aligned} \begin{aligned} v_2(\zeta , {\Im })=e^{-{\Im }}\sin (\zeta ). \end{aligned} \end{aligned}$$

By applying Eqs. (56) and (57) and the Aboodh Transform (AT) on Eqs. (54) and (55), we are able to derive:

60 $$\begin{aligned} \begin{aligned}{}&v_1(\zeta , {\Im })-\frac{\sin (\zeta )}{s^2}-\frac{1}{s^p}\Bigg [\frac{\partial ^2 v_{1}(\zeta ,{\Im })}{\partial \zeta ^2}\Bigg ]-\frac{2}{s^p}\mathcal {A}_{{\Im }}\Bigg [\mathcal {A}_{{\Im }}^{-1}v_{1}(\zeta ,{\Im })\frac{\partial \mathcal {A}_{{\Im }}^{-1}v_{1}(\zeta ,{\Im })}{\partial \zeta }\Bigg ]\\ {}&\quad +\frac{1}{s^p}\mathcal {A}_{{\Im }}\Bigg [\mathcal {A}_{{\Im }}^{-1}v_{2}(\zeta ,{\Im })\frac{\partial \mathcal {A}_{{\Im }}^{-1}v_{1}(\zeta ,{\Im })}{\partial \zeta }\Bigg ]+\frac{1}{s^p}\mathcal {A}_{{\Im }}\Bigg [\mathcal {A}_{{\Im }}^{-1}v_{1}(\zeta ,{\Im })\frac{\partial \mathcal {A}_{{\Im }}^{-1}v_{2}(\zeta ,{\Im })}{\partial \zeta }\Bigg ]=0, \end{aligned} \end{aligned}$$

61 $$\begin{aligned} \begin{aligned}{}&v_2(\zeta , {\Im })-\frac{\sin (\zeta )}{s^2}-\frac{1}{s^p}\Bigg [\frac{\partial ^2 v_{2}(\zeta ,{\Im })}{\partial \zeta ^2}\Bigg ]-\frac{2}{s^p}\mathcal {A}_{{\Im }}\Bigg [\mathcal {A}_{{\Im }}^{-1}v_{2}(\zeta ,{\Im })\frac{\partial \mathcal {A}_{{\Im }}^{-1}v_{2}(\zeta ,{\Im })}{\partial \zeta }\Bigg ]\\ {}&\quad+\frac{1}{s^p}\mathcal {A}_{{\Im }}\Bigg [\mathcal {A}_{{\Im }}^{-1}v_{2}(\zeta ,{\Im })\frac{\partial \mathcal {A}_{{\Im }}^{-1}v_{1}(\zeta ,{\Im })}{\partial \zeta }\Bigg ]+\frac{1}{s^p}\mathcal {A}_{{\Im }}\Bigg [\mathcal {A}_{{\Im }}^{-1}v_{1}(\zeta ,{\Im })\frac{\partial \mathcal {A}_{{\Im }}^{-1}v_{2}(\zeta ,{\Im })}{\partial \zeta }\Bigg ]=0, \end{aligned} \end{aligned}$$

The $k^{th}$ truncated term series are

62 $$\begin{aligned} \begin{aligned} v_1({\zeta },s)=\frac{\sin (\zeta )}{s^2}+\sum _{r=1}^{k}\frac{f_{r}({\zeta },s)}{s^{rp+1}}, \ \ r=1,2,3,4\ldots . \end{aligned} \end{aligned}$$

63 $$\begin{aligned} \begin{aligned} v_2({\zeta },s)=\frac{\sin (\zeta )}{s^2}+\sum _{r=1}^{k}\frac{g_{r}({\zeta },s)}{s^{rp+1}}, \ \ r=1,2,3,4\ldots . \end{aligned} \end{aligned}$$

Aboodh residual functions (ARFs) are

64 $$\begin{aligned} \begin{aligned}{}&\mathcal {A}_{{{\Im }}}Res({\zeta },s)=v_1(\zeta , {\Im })-\frac{\sin (\zeta )}{s^2}-\frac{1}{s^p}\Bigg [\frac{\partial ^2 v_{1}(\zeta ,{\Im })}{\partial \zeta ^2}\Bigg ]-\frac{1}{s^p}\mathcal {A}_{{\Im }}\Bigg [\mathcal {A}_{{\Im }}^{-1}v_{1}(\zeta ,{\Im })\frac{\partial \mathcal {A}_{{\Im }}^{-1}v_{1}(\zeta ,{\Im })}{\partial \zeta }\Bigg ]\\ {}&\quad \quad \quad \quad \quad \quad \quad +\frac{1}{s^p}\mathcal {A}_{{\Im }}\Bigg [\mathcal {A}_{{\Im }}^{-1}v_{2}(\zeta ,{\Im })\frac{\partial \mathcal {A}_{{\Im }}^{-1}v_{1}(\zeta ,{\Im })}{\partial \zeta }\Bigg ]+\frac{1}{s^p}\mathcal {A}_{{\Im }}\Bigg [\mathcal {A}_{{\Im }}^{-1}v_{1}(\zeta ,{\Im })\frac{\partial \mathcal {A}_{{\Im }}^{-1}v_{2}(\zeta ,{\Im })}{\partial \zeta }\Bigg ]=0, \end{aligned} \end{aligned}$$

65 $$\begin{aligned} \begin{aligned}{}&\mathcal {A}_{{{\Im }}}Res({\zeta },s)=v_2(\zeta , {\Im })-\frac{\sin (\zeta )}{s^2}-\frac{1}{s^p}\Bigg [\frac{\partial ^2 v_{2}(\zeta ,{\Im })}{\partial \zeta ^2}\Bigg ]-\frac{2}{s^p}\mathcal {A}_{{\Im }}\Bigg [\mathcal {A}_{{\Im }}^{-1}v_{2}(\zeta ,{\Im })\frac{\partial \mathcal {A}_{{\Im }}^{-1}v_{2}(\zeta ,{\Im })}{\partial \zeta }\Bigg ]\\ {}&\quad \quad \quad \quad \quad \quad \quad +\frac{1}{s^p}\mathcal {A}_{{\Im }}\Bigg [\mathcal {A}_{{\Im }}^{-1}v_{2}(\zeta ,{\Im })\frac{\partial \mathcal {A}_{{\Im }}^{-1}v_{1}(\zeta ,{\Im })}{\partial \zeta }\Bigg ]+\frac{1}{s^p}\mathcal {A}_{{\Im }}\Bigg [\mathcal {A}_{{\Im }}^{-1}v_{1}(\zeta ,{\Im })\frac{\partial \mathcal {A}_{{\Im }}^{-1}v_{2}(\zeta ,{\Im })}{\partial \zeta }\Bigg ]=0, \end{aligned} \end{aligned}$$

and the ${k}^{th}$-LRFs as:

66 $$\begin{aligned} \begin{aligned}{}&\mathcal {A}_{{{\Im }}}Res_{k}({\zeta },s)={v_1}_{k}(\zeta , {\Im })-\frac{\sin (\zeta )}{s^2}-\frac{1}{s^p}\Bigg [\frac{\partial ^2 {v_{1}}_{k}(\zeta ,{\Im })}{\partial \zeta ^2}\Bigg ]-\frac{1}{s^p}\mathcal {A}_{{\Im }}\Bigg [\mathcal {A}_{{\Im }}^{-1}{v_{1}}_{k}(\zeta ,{\Im })\frac{\partial \mathcal {A}_{{\Im }}^{-1}{v_{1}}_{k}(\zeta ,{\Im })}{\partial \zeta }\Bigg ]\\ {}&\quad \quad \quad \quad \quad \quad \quad +\frac{1}{s^p}\mathcal {A}_{{\Im }}\Bigg [\mathcal {A}_{{\Im }}^{-1}{v_{2}}_{k}(\zeta ,{\Im })\frac{\partial \mathcal {A}_{{\Im }}^{-1}{v_{1}}_{k}(\zeta ,{\Im })}{\partial \zeta }\Bigg ]+\frac{1}{s^p}\mathcal {A}_{{\Im }}\Bigg [\mathcal {A}_{{\Im }}^{-1}{v_{1}}_{k}(\zeta ,{\Im })\frac{\partial \mathcal {A}_{{\Im }}^{-1}{v_{2}}_{k}(\zeta ,{\Im })}{\partial \zeta }\Bigg ]=0, \end{aligned} \end{aligned}$$

67 $$\begin{aligned} \begin{aligned}{}&\mathcal {A}_{{{\Im }}}Res_{k}({\zeta },s)={v_2}_{k}(\zeta , {\Im })-\frac{\sin (\zeta )}{s^2}-\frac{1}{s^p}\Bigg [\frac{\partial ^2 {v_{2}}_{k}(\zeta ,{\Im })}{\partial \zeta ^2}\Bigg ]-\frac{1}{s^p}\mathcal {A}_{{\Im }}\Bigg [\mathcal {A}_{{\Im }}^{-1}{v_{2}}_{k}(\zeta ,{\Im })\frac{\partial \mathcal {A}_{{\Im }}^{-1}{v_{2}}_{k}(\zeta ,{\Im })}{\partial \zeta }\Bigg ]\\ {}&\quad \quad \quad \quad \quad \quad \quad +\frac{1}{s^p}\mathcal {A}_{{\Im }}\Bigg [\mathcal {A}_{{\Im }}^{-1}{v_{2}}_{k}(\zeta ,{\Im })\frac{\partial \mathcal {A}_{{\Im }}^{-1}{v_{1}}_{k}(\zeta ,{\Im })}{\partial \zeta }\Bigg ]+\frac{1}{s^p}\mathcal {A}_{{\Im }}\Bigg [\mathcal {A}_{{\Im }}^{-1}{v_{1}}_{k}(\zeta ,{\Im })\frac{\partial \mathcal {A}_{{\Im }}^{-1}{v_{2}}_{k}(\zeta ,{\Im })}{\partial \zeta }\Bigg ]=0, \end{aligned} \end{aligned}$$

To calculate $f_{r}({\zeta },s)$ and $g_{r}({\zeta },s)$, do the following procedures: The $r^{th}$-truncated series from Eqs. (62) and (63) should be substituted into the $r^{th}$-Aboodh residual function depicted in Eqs. (66) and (67), and the resultant equations should be multiplied by $s^{r{{\varrho }}+1}$. The relations $\lim _{s\rightarrow \infty }(s^{r{{\varrho }}+1} {A}{{{\Im }}}Res{v_{1},r}({\zeta },s))=0$ and $\lim _{s\rightarrow \infty }(s^{r{{\varrho }}+1} {A}{{{\Im }}}Res{v_{2},r}({\zeta },s))=0$ are then solved iteratively.in the case of $r = 1, 2, 3, \ldots$. The following are the first few terms:

68 $$\begin{aligned} \begin{aligned}{}&f_{1}({\zeta },s)=-\sin (\zeta ),\\&g_{1}({\zeta },s)=-\sin (\zeta ), \end{aligned} \end{aligned}$$

69 $$\begin{aligned} \begin{aligned}{}&f_{2}(\zeta ,s)=\sin (\zeta ),\\&g_{2}(\zeta ,s)=\sin (\zeta ). \end{aligned} \end{aligned}$$

70 $$\begin{aligned} \begin{aligned}{}&f_{2}(\zeta ,s)=-\sin (\zeta ),\\&g_{2}(\zeta ,s)=-\sin (\zeta ). \end{aligned} \end{aligned}$$

and so on.

Substituted $f_{r}({\zeta },s)$ and $g_{r}({\zeta },s)$ for $r=1,2,3,\ldots ,$ in Eqs. (62), (63), we get

71 $$\begin{aligned} \begin{aligned}{}&v_1(\zeta ,s)=-\frac{\sin (\zeta )}{s^{p +1}}+\frac{\sin (\zeta )}{s^{2 p +1}}-\frac{\sin (\zeta )}{s^{3p +1}}+\frac{\sin (\zeta )}{s}+\cdots .\\ \end{aligned} \end{aligned}$$

72 $$\begin{aligned} \begin{aligned}{}&v_2(\zeta ,s)=-\frac{\sin (\zeta )}{s^{p +1}}+\frac{\sin (\zeta )}{s^{2 p +1}}-\frac{\sin (\zeta )}{s^{3p +1}}+\frac{\sin (\zeta )}{s}+\cdots .\\ \end{aligned} \end{aligned}$$

Applying the inverse transform of Aboodh yields

73 $$\begin{aligned} \begin{aligned}{}&v_1(\zeta ,{\Im })=\sin (\zeta )+\frac{\sin (\zeta ) {\Im } ^{2 p}}{\Gamma (2 p+1)}-\frac{\sin (\zeta ) {\Im } ^{3 p}}{\Gamma (3 p+1)}-\frac{\sin (\zeta ) {\Im } ^p}{\Gamma (p+1)}+\cdots .\\ \end{aligned} \end{aligned}$$

74 $$\begin{aligned} \begin{aligned}{}&v_2(\zeta ,{\Im })=\sin (\zeta )+\frac{\sin (\zeta ) {\Im } ^{2 p}}{\Gamma (2 p+1)}-\frac{\sin (\zeta ) {\Im } ^{3 p}}{\Gamma (3 p+1)}-\frac{\sin (\zeta ) {\Im } ^p}{\Gamma (p+1)}+\cdots .\\ \end{aligned} \end{aligned}$$

**Figure 3:**
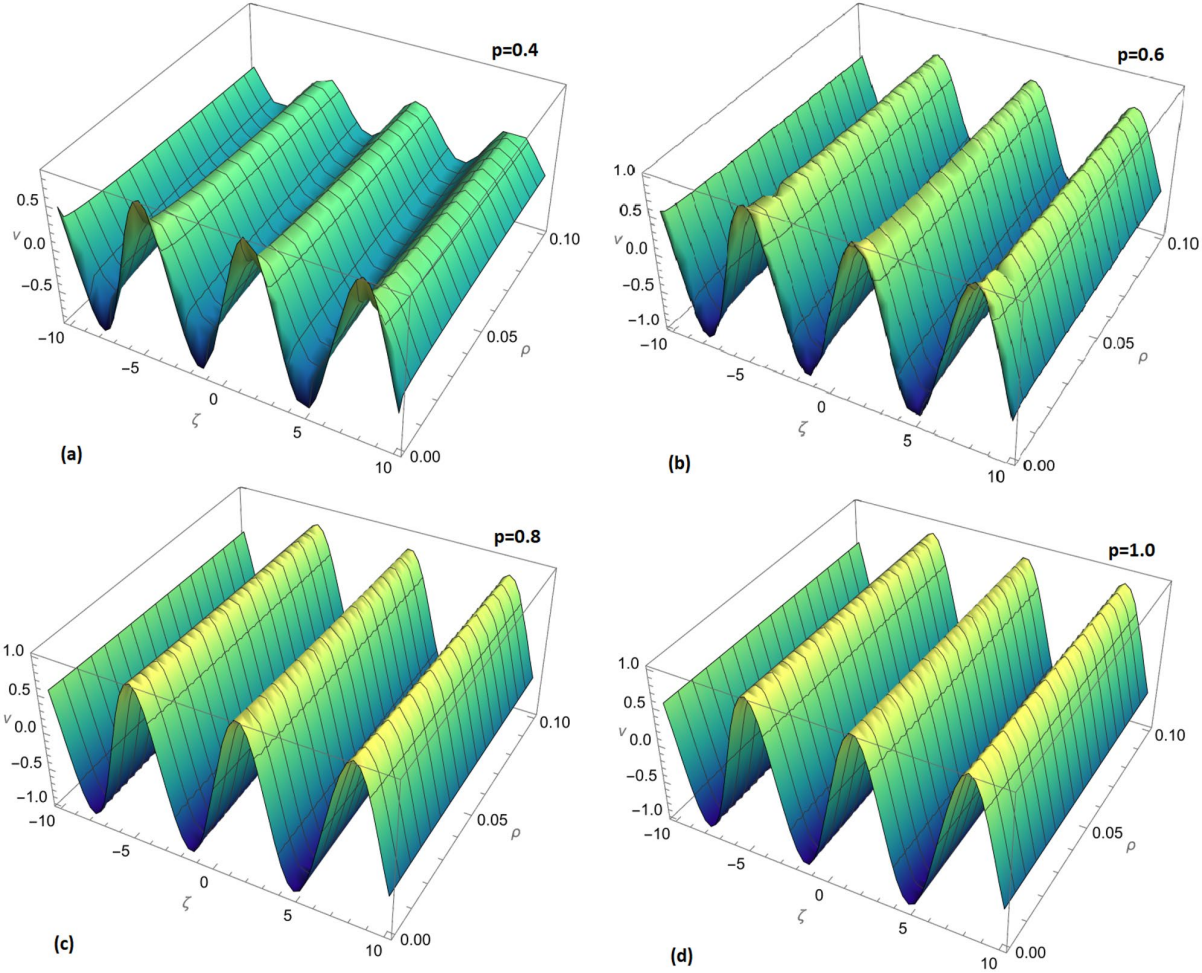
Fractional order comparison of $v_{1}(\zeta ,{\Im })$ and $v_{2}(\zeta ,{\Im })$ for ${\Im }=0.1$.

**Figure 4:**
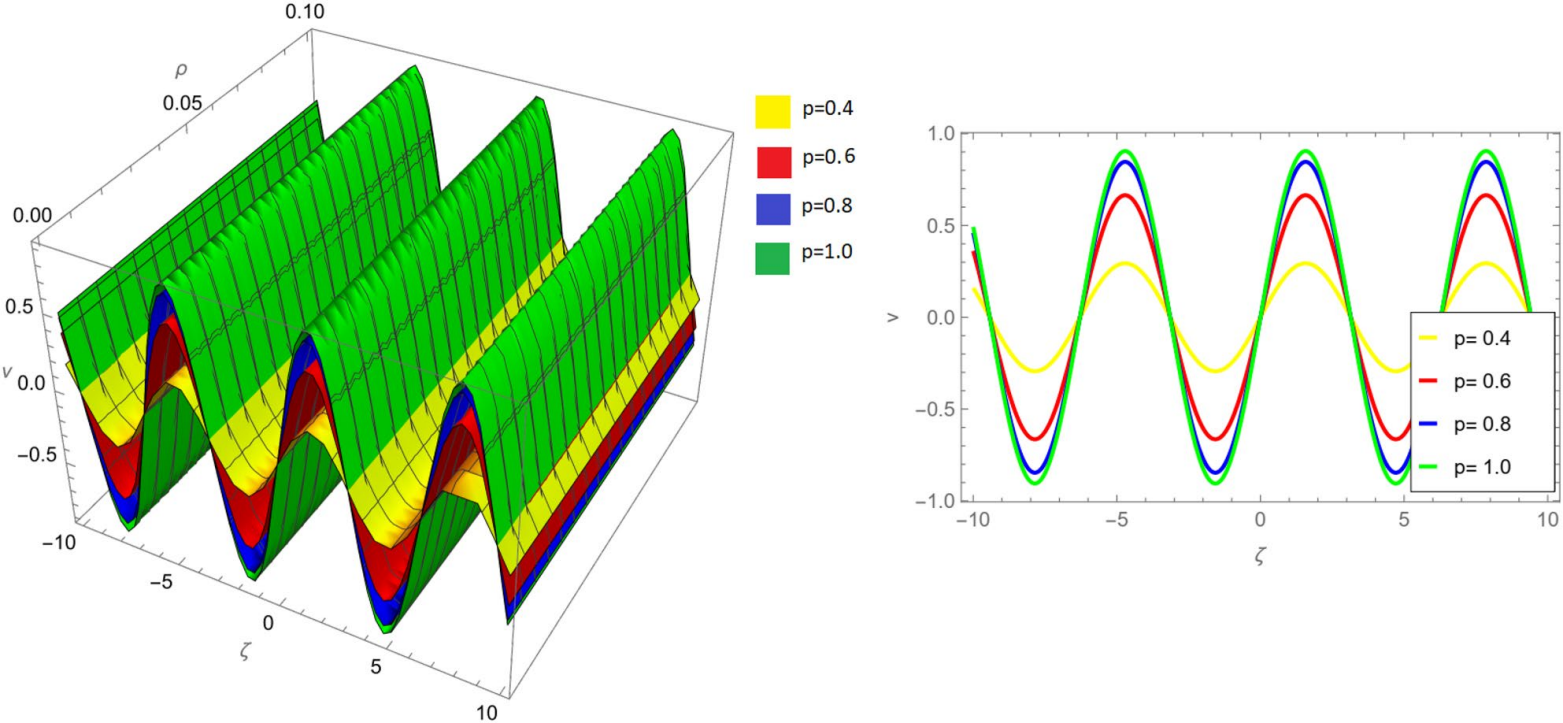
Fractional order 3D and 2D comparison of $v_{1}(\zeta ,{\Im })$ and $v_{2}(\zeta ,{\Im })$ for ${\Im }=0.1$.

**Table 2:** Analysis of the ARPSM solution for various fractional order of example 2 for $v_{1}(\zeta ,{\Im })$ and $v_{2}(\zeta ,{\Im })$ for ${\Im }=0.1$.

$\zeta$	$ARPSM_{P=0.6}$	$ARPSM_{p=0.8}$	$ARPSM_{P=1.0}$	Exact	Error of $p=0.6$	Error of $p=0.8$	Error of $p=1.0$
− 0.5	− 0.36757	− 0.405627	− 0.4338	− 0.433802	0.0662322	0.0281749	1.958310 $\times\,10^{-6}$
− 0.4	− 0.298562	− 0.329475	− 0.352359	− 0.35236	0.0537978	0.0228853	1.590658 $\times\,10^{-6}$
− 0.3	− 0.226572	− 0.250031	− 0.267397	− 0.267398	0.0408259	0.0173671	1.207112 $\times\,10^{-6}$
− 0.2	− 0.152317	− 0.168088	− 0.179763	− 0.179763	0.027446	0.0116754	8.115051 $\times\,10^{-7}$
− 0.1	− 0.0765411	− 0.084466	− 0.0903326	− 0.090333	0.0137919	0.00586701	4.077898 $\times\,10^{-7}$
0.1	0.0765411	0.084466	0.0903326	0.090333	0.0137919	0.00586701	4.077898 $\times\,10^{-7}$
0.2	0.152317	0.168088	0.179763	0.179763	0.027446	0.0116754	8.115051 $\times\,10^{-7}$
0.3	0.226572	0.250031	0.267397	0.267398	0.0408259	0.0173671	1.207112 $\times\,10^{-6}$
0.4	0.298562	0.329475	0.352359	0.35236	0.0537978	0.0228853	1.590658 $\times\,10^{-6}$
0.5	0.36757	0.405627	0.4338	0.433802	0.0662322	0.0281749	1.958310 $\times\,10^{-6}$

Table 1 presents a comprehensive analysis of the Aboodh Residual Power Series Method (ARPSM) solution for various fractional orders of example 1, specifically for $v_{1}(\zeta ,{\Im })$ at ${\Im }=0.1$. The table provides valuable insights into the behavior of the solution under different fractional orders, allowing for a detailed examination of its performance.

In Fig. 1, a visual comparison is made between the exact solution and the approximate solution of $v_{1}(\zeta ,{\Im })$ for ${\Im }=0.1$. The graph highlights the accuracy of the ARPSM method in capturing the dynamics of the solution, showcasing a close match between the two.

Figure 2 extends the comparison further by presenting both the exact and approximate solutions of $v_{1}(\zeta ,{\Im })$ in subgraph (a). Subgraph (b) offers a fractional order comparison, providing a detailed view of how the solution behaves under different fractional orders at ${\Im }=0.1$. This comprehensive graphical representation aids in understanding the method’s sensitivity to variations in fractional orders.

Moving to Fig. 3, a fractional order comparison is presented not only for $v_{1}(\zeta ,{\Im })$ but also for $v_{2}(\zeta ,{\Im })$ at ${\Im }=0.1$. This comparison allows for an assessment of the method’s consistency in handling multiple variables within the solution space.

Figure 4 takes the analysis further by providing both 3D and 2D fractional order comparisons of $v_{2}(\zeta ,{\Im })$ and $v_{2}(\zeta ,{\Im })$ at ${\Im }=0.1$. These visual representations offer a more intricate understanding of the solution’s behavior, emphasizing the method’s efficacy in capturing the solution’s complexities.

Table 2 follows a similar structure to Table 1, providing a detailed analysis of the ARPSM solution for various fractional orders of example 2, specifically for $v_{1}(\zeta ,{\Im })$ and $v_{2}(\zeta ,{\Im })$ at ${\Im }=0.1$. This comparative analysis, both in tabular and graphical formats, contributes to a thorough evaluation of the ARPSM method’s performance in solving the given examples under different fractional orders.

### The core idea behind the Aboodh transform iterative method

Below is the fractional PDE for both space and time.

75 $$\begin{aligned} D_{{{\Im }}}^{{{\varrho }}}{{v}}({\zeta },{{\Im }})=\Phi \Big ({{v}}({\zeta },{{\Im }}),D_{{\zeta }}^{v}{{v}}({\zeta },{{\Im }}),D_{{\zeta }}^{2v}{{v}}({\zeta },{{\Im }}),D_{{\zeta }}^{3v}{{v}}({\zeta },{{\Im }})\Big ),\ 0<{{\varrho }},v\le 1, \end{aligned}$$

with the IC’s

76 $$\begin{aligned} {{v}}^{(k)}({\zeta },0)=h_{k},\ k=0,1,2,\ldots ,m-1, \end{aligned}$$

${{v}}({\zeta },{{\Im }})$ is the function which is unkhown and $\Phi \Big ({{v}}({\zeta },{{\Im }}),D_{{\zeta }}^{v}{{v}}({\zeta },{{\Im }}),D_{{\zeta }}^{2v}{{v}}({\zeta },{{\Im }}),D_{{\zeta }}^{3v}{{v}}({\zeta },{{\Im }})\Big )$ can be linear or nonlinear operator of ${{v}}({\zeta },{{\Im }}),D_{{\zeta }}^{v}{{v}}({\zeta },{{\Im }}),D_{{\zeta }}^{2v}{{v}}({\zeta },{{\Im }})$ and $D_{{\zeta }}^{3v}{{v}}({\zeta },{{\Im }})$. We use *v* to conveniently express ${{v}}({\zeta }, {{\Im }})$. As a result, by applying the AT to both sides of Eq. 75, we get the following equation:

77 $$\begin{aligned} {A} [{{v}}({\zeta },{{\Im }})]=\frac{1}{{s}^{{{\varrho }}}}\left( \sum _{k=0}^{m-1}\frac{{{v}}^{(k)}({\zeta },0)}{{s}^{2-{{\varrho }}+k}}+{A} \left[ \Phi \Big ({{v}}({\zeta },{{\Im }}),D_{{\zeta }}^{v}{{v}}({\zeta },{{\Im }}),D_{{\zeta }}^{2v}{{v}}({\zeta },{{\Im }}),D_{{\zeta }}^{3v}{{v}}({\zeta },{{\Im }})\Big )\right] \right) , \end{aligned}$$

After applying the inverse Aboodh transformation, the following equation is obtained:

78 $$\begin{aligned} {{v}}({\zeta },{{\Im }})={A}^{-1}\left[ \frac{1}{{s}^{{{\varrho }}}}\left( \sum _{k=0}^{m-1}\frac{{{v}}^{(k)}({\zeta },0)}{{s}^{2-{{\varrho }}+k}}+{A} \Big [\Phi \Big ({{v}}({\zeta },{{\Im }}),D_{{\zeta }}^{v}{{v}}({\zeta },{{\Im }}),D_{{\zeta }}^{2v}{{v}}({\zeta },{{\Im }}),D_{{\zeta }}^{3v}{{v}}({\zeta },{{\Im }})\Big )\Big ]\right) \right] . \end{aligned}$$

By applying the Aboodh transform iteratively, an infinite series is produced as the solution.

79 $$\begin{aligned} {{v}}({\zeta },{{\Im }})=\sum _{i=0}^{\infty }{{v}}_{i}. \end{aligned}$$

In this instance, $\Phi \Big ({{v}},D_{{\zeta }}^{v}{{v}},D_{{\zeta }}^{2v}{{v}},D_{{\zeta }}^{3v}{{v}}\Big )$ be the operator, which may be either linear or nonlinear, can be decomposed into the following:

80 $$\begin{aligned} \begin{aligned}{}&\Phi \Big ({{v}},D_{{\zeta }}^{v}{{v}},D_{{\zeta }}^{2v}{{v}},D_{{\zeta }}^{3v}{{v}}\Big )=\Phi \Big ({{v}}_{0},D_{{\zeta }}^{v}{{v}}_{0},D_{{\zeta }}^{2v}{{v}}_{0},D_{{\zeta }}^{3v}{{v}}_{0}\Big )\\ {}&\quad \quad \quad \quad \quad \quad \quad \quad \quad \quad \quad \quad \;+ \sum _{i=0}^{\infty } \left( \Phi \left( \sum _{k=0}^{i}\Big ({{v}}_{k},D_{{\zeta }}^{v}{{v}}_{k},D_{{\zeta }}^{2v}{{v}}_{k},D_{{\zeta }}^{3v}{{v}}_{k}\Big )\right) -\Phi \left( \sum _{k=1}^{i-1}\Big ({{v}}_{k},D_{{\zeta }}^{v}{{v}}_{k},D_{{\zeta }}^{2v}{{v}}_{k},D_{{\zeta }}^{3v}{{v}}_{k}\Big )\right) \right) . \end{aligned} \end{aligned}$$

After inserting Eqs. (80) and 79 into Eq. (78), the following equation is produced:

81 $$\begin{aligned} \begin{aligned}{}&\sum _{i=0}^{\infty }{{v}}_i({\zeta },{{\Im }})={A}^{-1}\left[ \frac{1}{{s}^{{{\varrho }}}}\left( \sum _{k=0}^{m-1}\frac{{{v}}^{(k)}({\zeta },0)}{{s}^{2-{{\varrho }}+k}}+{A}\big [\Phi ({{v}}_{0},D_{{\zeta }}^{v}{{v}}_{0},D_{{\zeta }}^{2v}{{v}}_{0},D_{{\zeta }}^{3v}{{v}}_{0})\big ]\right) \right] \\ {}&\quad \quad \quad \quad \quad \quad \quad +{A}^{-1}\left[ \frac{1}{{s}^{{{\varrho }}}}\left( {A}\left[ \sum _{i=0}^{\infty }\left( \Phi \sum _{k=0}^{i}\big ({{v}}_{k},D_{{\zeta }}^{v}{{v}}_{k},D_{{\zeta }}^{2v}{{v}}_{k},D_{{\zeta }}^{3v}{{v}}_{k}\big )\right) \right] \right) \right] \\ {}&\quad \quad \quad \quad \quad \quad \quad -{A}^{-1}\left[ \frac{1}{{s}^{{{\varrho }}}}\left( {A}\left[ \left( \Phi \sum _{k=1}^{i-1}\big ({{v}}_{k},D_{{\zeta }}^{v}{{v}}_{k},D_{{\zeta }}^{2v}{{v}}_{k},D_{{\zeta }}^{3v}{{v}}_{k}\big )\right) \right] \right) \right] \end{aligned} \end{aligned}$$

82 $$\begin{aligned} \begin{aligned}{}&{{v}}_0({\zeta },{{\Im }})={A}^{-1}\left[ \frac{1}{{s}^{{{\varrho }}}}\left( \sum _{k=0}^{m-1}\frac{{{v}}^{(k)}({\zeta },0)}{{s}^{2-{{\varrho }}+k}}\right) \right] ,\\&{{v}}_1({\zeta },{{\Im }})={A}^{-1}\Big [ \frac{1}{{s}^{{{\varrho }}}}\Big ( {A}\big [\Phi ({{v}}_{0},D_{{\zeta }}^{v}{{v}}_{0},D_{{\zeta }}^{2v}{{v}}_{0},D_{{\zeta }}^{3v}{{v}}_{0})\big ]\Big )\Big ],\\ \vdots \\&{{v}}_{m+1}({\zeta },{{\Im }})={A}^{-1}\left[ \frac{1}{{s}^{{{\varrho }}}}\left( {A}\left[ \sum _{i=0}^{\infty }\left( \Phi \sum _{k=0}^{i}({{v}}_{k},D_{{\zeta }}^{v}{{v}}_{k},D_{{\zeta }}^{2v}{{v}}_{k},D_{{\zeta }}^{3v}{{v}}_{k})\right) \right] \right) \right] \\ {}&\quad \quad \quad \quad \quad \quad \quad -{A}^{-1}\left[ \frac{1}{{s}^{{{\varrho }}}}\left( {A}\left[ \left( \Phi \sum _{k=1}^{i-1}({{v}}_{k},D_{{\zeta }}^{v}{{v}}_{k},D_{{\zeta }}^{2v}{{v}}_{k},D_{{\zeta }}^{3v}{{v}}_{k})\right) \right] \right) \right] ,\ m=1,2,\cdots . \end{aligned} \end{aligned}$$

Eq. (75) yields the m-term solution, which may be found analytically as follows:

83 $$\begin{aligned} {{v}}({\zeta },{{\Im }})=\sum _{i=0}^{m-1}{{v}}_{i}. \end{aligned}$$

#### Problem with NITM

#### Problem 1

84 $$\begin{aligned} \begin{aligned}{}&D_{{\Im }}^{{\varrho }} v_{1}(\zeta , {\Im })=-6v_{1}^{2}(\zeta ,{\Im })\frac{\partial v_{1}(\zeta ,{\Im })}{\partial \zeta }-\frac{\partial ^{3} v_{1}(\zeta ,{\Im })}{\partial \zeta ^{3}}, \ \text{ where } \ \ 0<p \le 1 \end{aligned} \end{aligned}$$

Having the IC’s:

85 $$\begin{aligned} \begin{aligned} v_{1}(\zeta , 0)=\sqrt{c}\ \text {sech}\left( k+\sqrt{c} \zeta \right) , \end{aligned} \end{aligned}$$

The following equations develop when both sides of Eq. (84) are subjected to the Aboodh transform:

86 $$\begin{aligned} \begin{aligned}{}&{A} [D_{{\Im }}^{{\varrho }} v_{1}(\zeta , {\Im })]=\frac{1}{{s}^{{{\varrho }}}}\left( \sum _{k=0}^{m-1}\frac{v_{1}^{(k)}({\zeta },0)}{{s}^{2-{{\varrho }}+k}} +{A}\Big [-6v_{1}^{2}(\zeta ,{\Im })\frac{\partial v_{1}(\zeta ,{\Im })}{\partial \zeta }-\frac{\partial ^{3} v_{1}(\zeta ,{\Im })}{\partial \zeta ^{3}}\Big ]\right) \\ \end{aligned} \end{aligned}$$

The following equations result from applying the inverse Aboodh transform to Eq. (86):

87 $$\begin{aligned} \begin{aligned}{}&v_{1}(\zeta , {\Im })=A^{-1}\left[ \frac{1}{{s}^{{{\varrho }}}}\left( \sum _{k=0}^{m-1}\frac{v_{1}^{(k)}({\zeta },0)}{{s}^{2-{{\varrho }}+k}} +{A}\Big [-6v_{1}^{2}(\zeta ,{\Im })\frac{\partial v_{1}(\zeta ,{\Im })}{\partial \zeta }-\frac{\partial ^{3} v_{1}(\zeta ,{\Im })}{\partial \zeta ^{3}}\Big ]\right) \right] \\ \end{aligned} \end{aligned}$$

The following expression is obtained by using the Aboodh transform iteratively:

$$\begin{aligned} \begin{aligned}{}&(v_1)_{0}(\zeta , {\Im })={A}^{-1}\left[ \frac{1}{{s}^{{{\varrho }}}}\left( \sum _{k=0}^{m-1}\frac{v_{1}^{(k)}({\zeta },0)}{{s}^{2-{{\varrho }}+k}}\right) \right] \\&\quad \quad \quad \quad \quad ={A}^{-1}\Big [\frac{v_{1}({\zeta },0)}{{s}^2}\Big ]\\&\quad \quad \quad \quad \quad =\sqrt{c}\ \text {sech}\left( k+\sqrt{c} \zeta \right) , \end{aligned} \end{aligned}$$

RL integral is applied to Eq. (84) to get the equivalent form.

88 $$\begin{aligned} \begin{aligned}{}&v_{1}(\zeta , {\Im })=\sqrt{c}\ \text {sech}\left( k+\sqrt{c} \zeta \right) +{A}\Bigg [-6v_{1}^{2}(\zeta ,{\Im })\frac{\partial v_{1}(\zeta ,{\Im })}{\partial \zeta }-\frac{\partial ^{3} v_{1}(\zeta ,{\Im })}{\partial \zeta ^{3}}\Bigg ]\\ \end{aligned} \end{aligned}$$

The terms that come from the NITM procedure are as follows.

89 $$\begin{aligned} \begin{aligned}{}&{v_{1}}_{0}(\zeta ,{\Im })=\sqrt{c}\ \text {sech}\left( k+\sqrt{c} \zeta \right) ,\\&{v_{1}}_{1}(\zeta ,{\Im })=\frac{c^2 {\Im } ^p \tanh \left( \zeta \sqrt{c}+k\right) \text {sech}\left( \zeta \sqrt{c}+k\right) }{\Gamma (p+1)},\\&{v_{1}}_{2}(\zeta ,{\Im })=\frac{c^{7/2} {\Im } ^{2 p} \Big (\cosh \Big (2 \Big (\zeta \sqrt{c}+k\Big )\Big )-3\Big ) \text {sech}^3\Big (\zeta \sqrt{c}+k\Big )}{2 \Gamma (2 p+1)}\\ {}&\quad \quad \quad \quad \quad \quad +\Big (3 c^5 {\Im } ^{3 p} \tanh \Big (\zeta \sqrt{c}+k\Big ) \text {sech}^5\Big (\zeta \sqrt{c}+k\Big )\times \Big (c^{3/2} {\Im } ^p \Gamma (3 p+1)^2\Big (\cosh \Big (2 \Big (\zeta \sqrt{c}+k\Big )\Big )-3\Big ) \tanh \Big (\zeta \sqrt{c}+k\Big )\\ {}&\quad \quad \quad \quad \quad \quad +\Gamma (p+1) \Gamma (2 p+1) \Gamma (4 p+1) \Big (3 \cosh \Big (2 (\zeta \sqrt{c}+k)\Big )-7\Big )\Big )/\Big (\Gamma (p+1)^3 \Gamma (3 p+1) \Gamma (4 p+1)\Big ).\\ \end{aligned} \end{aligned}$$

The NITM algorithm’s final result is under

90 $$\begin{aligned} \begin{aligned} v_{1}(\zeta ,{\Im })={v_{1}}_{0}(\zeta ,{\Im })+{v_{1}}_{1}(\zeta ,{\Im })+{v_{1}}_{2}(\zeta ,{\Im })+\cdots . \end{aligned} \end{aligned}$$

91 $$\begin{aligned} \begin{aligned}{}&v_1(\zeta ,{\Im })=\sqrt{c}\ \text {sech}\left( k+\sqrt{c} \zeta \right) +\frac{c^2 {\Im } ^p \tanh \left( \zeta \sqrt{c}+k\right) \text {sech}\left( \zeta \sqrt{c}+k\right) }{\Gamma (p+1)}\\ {}&\quad \quad \quad \quad \quad \; +\frac{c^{7/2} {\Im } ^{2 p} \Big (\cosh \Big (2 \Big (\zeta \sqrt{c}+k\Big )\Big )-3\Big ) \text {sech}^3\Big (\zeta \sqrt{c}+k\Big )}{2 \Gamma (2 p+1)}\\ {}&\quad \quad \quad \quad \quad \;+\Big (3 c^5 {\Im } ^{3 p} \tanh \Big (\zeta \sqrt{c}+k\Big ) \text {sech}^5\Big (\zeta \sqrt{c}+k\Big )\times \Big (c^{3/2} {\Im } ^p \Gamma (3 p+1)^2(\cosh \Big (2 \Big (\zeta \sqrt{c}+k\Big )\Big )-3\Big ) \tanh \Big (\zeta \sqrt{c}+k\Big )\\ {}&\quad \quad \quad \quad \quad \;+\Gamma (p+1) \Gamma (2 p+1) \Gamma (4 p+1) \Big (3 \cosh \Big (2 (\zeta \sqrt{c}+k)\Big )-7\Big )\Big )/\Big (\Gamma (p+1)^3 \Gamma (3 p+1) \Gamma (4 p+1)\Big )+\cdots .\\ \end{aligned} \end{aligned}$$

**Table 3:** Analysis of the NITM solution for various fractional order of example 1 for $v_{1}(\zeta ,{\Im })$ for ${\Im }=0.1$.

$\zeta$	$ARPSM_{P=0.6}$	$ARPSM_{p=0.8}$	$ARPSM_{P=1.0}$	Exact	Error of $p=0.6$	Error of $p=0.8$	Error of $p=1.0$
0.1	0.223567	0.223559	0.223549	0.223549	0.0000184111	0.0000104484	2.886385 $\times\,10^{-12}$
0.2	0.22342	0.223401	0.223379	0.223379	0.0000409168	0.0000217275	5.602018 $\times\,10^{-12}$
0.3	0.223161	0.223131	0.223098	0.223098	0.0000633205	0.0000329525	8.235134 $\times\,10^{-12}$
0.4	0.222792	0.222751	0.222707	0.222707	0.000085567	0.0000440957	1.074754 $\times\,10^{-11}$
0.5	0.222313	0.222261	0.222206	0.222206	0.000107602	0.0000551296	1.310312 $\times\,10^{-11}$
0.6	0.221726	0.221662	0.221596	0.221596	0.000129372	0.0000660279	1.526911 $\times\,10^{-11}$
0.7	0.221031	0.220957	0.22088	0.22088	0.000150825	0.0000767646	1.721631 $\times\,10^{-11}$
0.8	0.220231	0.220146	0.220059	0.220059	0.000171912	0.000087315	1.891950 $\times\,10^{-11}$
0.9	0.219327	0.219232	0.219135	0.219135	0.000192586	0.0000976553	2.035818 $\times\,10^{-11}$
1.	0.218322	0.218217	0.21811	0.21811	0.0002128	0.000107763	2.151645 $\times\,10^{-11}$

**Figure 5:**
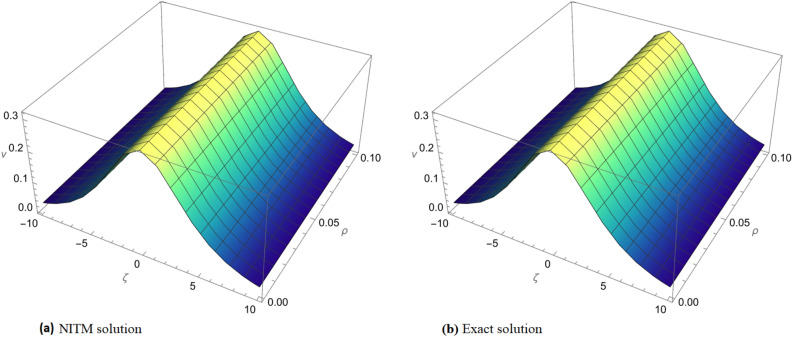
Comparison of the exact solution and approximate solution of $v_{1}(\zeta ,{\Im })$ for ${\Im }=0.1$.

**Figure 6:**
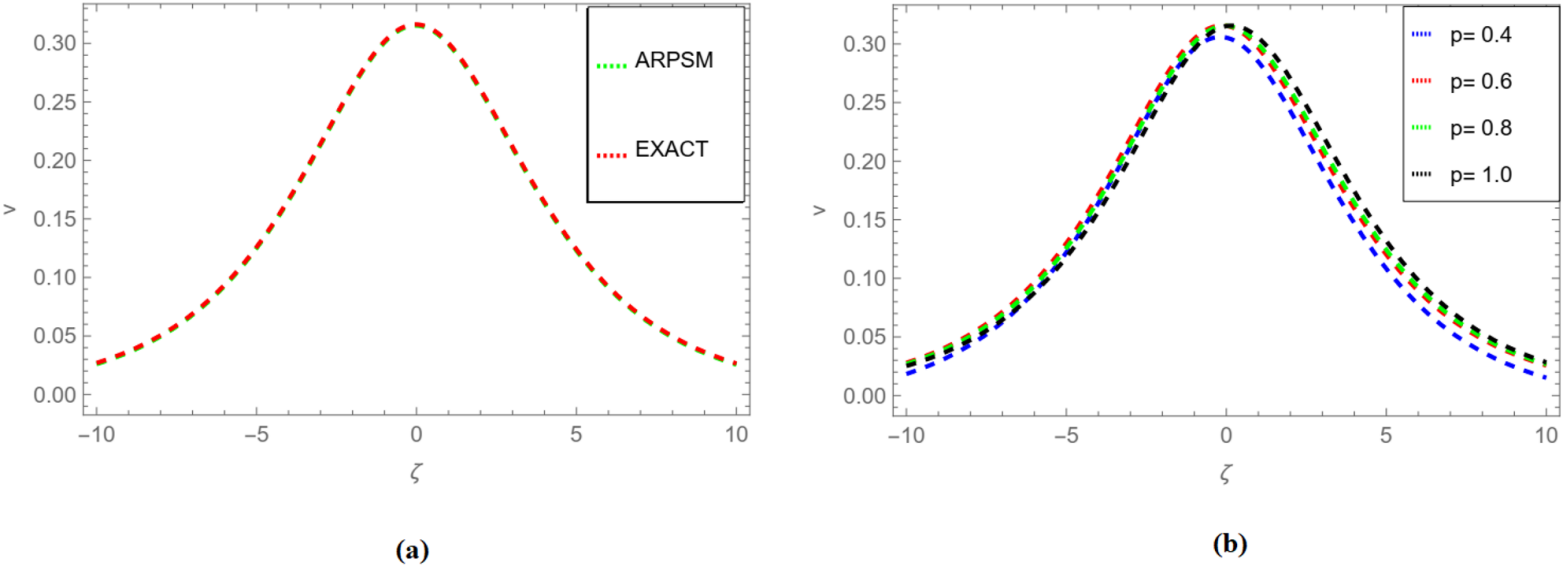
(**a**) Comparison of the exact solution and approximate solution. (**b**) Fractional order comparison of $v_{1}(\zeta ,{\Im })$ for ${\Im }=0.1$.

#### Problem 2

92 $$\begin{aligned} \begin{aligned}{}&D_{{\Im }}^{{\varrho }} v_{1}(\zeta , {\Im })=\frac{\partial ^2 v_{1}(\zeta ,{\Im })}{\partial \zeta ^2}+2v_{1}(\zeta ,{\Im })\frac{\partial v_{1}(\zeta ,{\Im })}{\partial \zeta }-v_{2}(\zeta ,{\Im })\frac{\partial v_{1}(\zeta ,{\Im })}{\partial \zeta }-v_{1}(\zeta ,{\Im })\frac{\partial v_{2}(\zeta ,{\Im })}{\partial \zeta }, \end{aligned} \end{aligned}$$

93 $$\begin{aligned} \begin{aligned}{}&D_{{\Im }}^{{\varrho }} v_{2}(\zeta , {\Im })=\frac{\partial ^2 v_{2}(\zeta ,{\Im })}{\partial \zeta ^2}+2v_{2}(\zeta ,{\Im })\frac{\partial v_{2}(\zeta ,{\Im })}{\partial \zeta }-v_{2}(\zeta ,{\Im })\frac{\partial v_{1}(\zeta ,{\Im })}{\partial \zeta }-v_{1}(\zeta ,{\Im })\frac{\partial v_{2}(\zeta ,{\Im })}{\partial \zeta },\\ {}&\ \text{ where } \ \ 0<p \le 1 \end{aligned} \end{aligned}$$

Subjected to the IC’s:

94 $$\begin{aligned} \begin{aligned} v_1(\zeta , 0)=\sin (\zeta ), \end{aligned} \end{aligned}$$

95 $$\begin{aligned} \begin{aligned} v_2(\zeta , 0)=\sin (\zeta ), \end{aligned} \end{aligned}$$

These equations are the outcome of applying the AT to both sides of Eqs. (92) and (93).

96 $$\begin{aligned} \begin{aligned}{}&{A} [D_{{\Im }}^{{\varrho }} v_{1}(\zeta , {\Im })]=\frac{1}{{s}^{{{\varrho }}}}\Bigg (\sum _{k=0}^{m-1}\frac{v_{1}^{(k)}({\zeta },0)}{{s}^{2-{{\varrho }}+k}} +{A}\Bigg [\frac{\partial ^2 v_{1}(\zeta ,{\Im })}{\partial \zeta ^2}+2v_{1}(\zeta ,{\Im })\frac{\partial v_{1}(\zeta ,{\Im })}{\partial \zeta }-v_{2}(\zeta ,{\Im })\frac{\partial v_{1}(\zeta ,{\Im })}{\partial \zeta }\\ &\quad\quad\quad\quad\quad\quad\quad\;-v_{1}(\zeta ,{\Im })\frac{\partial v_{2}(\zeta ,{\Im })}{\partial \zeta }\Bigg ]\Bigg )\\ \end{aligned} \end{aligned}$$

97 $$\begin{aligned} \begin{aligned}{}&{A} [D_{{\Im }}^{{\varrho }} v_{2}(\zeta , {\Im })]=\frac{1}{{s}^{{{\varrho }}}}\Bigg (\sum _{k=0}^{m-1}\frac{v_{2}^{(k)}({\zeta },0)}{{s}^{2-{{\varrho }}+k}} +{A}\Bigg [\frac{\partial ^2 v_{2}(\zeta ,{\Im })}{\partial \zeta ^2}+2v_{2}(\zeta ,{\Im })\frac{\partial v_{2}(\zeta ,{\Im })}{\partial \zeta }-v_{2}(\zeta ,{\Im })\frac{\partial v_{1}(\zeta ,{\Im })}{\partial \zeta }\\ &\quad\quad\quad\quad\quad\quad\quad\;-v_{1}(\zeta ,{\Im })\frac{\partial v_{2}(\zeta ,{\Im })}{\partial \zeta }\Bigg ]\Bigg )\\ \end{aligned} \end{aligned}$$

The following equations are obtained by applying the IAT to Eqs. (96) and (97):

98 $$\begin{aligned} \begin{aligned}{}&v_{1}(\zeta , {\Im })=A^{-1}\Bigg [\frac{1}{{s}^{{{\varrho }}}}\Bigg (\sum _{k=0}^{m-1}\frac{v_{1}^{(k)}({\zeta },0)}{{s}^{2-{{\varrho }}+k}} +{A}\Bigg [\frac{\partial ^2 v_{1}(\zeta ,{\Im })}{\partial \zeta ^2}+2v_{1}(\zeta ,{\Im })\frac{\partial v_{1}(\zeta ,{\Im })}{\partial \zeta }-v_{2}(\zeta ,{\Im })\frac{\partial v_{1}(\zeta ,{\Im })}{\partial \zeta }\\ &\quad\quad\quad\quad\quad\quad\quad\;-v_{1}(\zeta ,{\Im })\frac{\partial v_{2}(\zeta ,{\Im })}{\partial \zeta }\Bigg ]\Bigg )\Bigg ]\\ \end{aligned} \end{aligned}$$

99 $$\begin{aligned} \begin{aligned}{}&v_{2}(\zeta , {\Im })=A^{-1}\Bigg [\frac{1}{{s}^{{{\varrho }}}}\Bigg (\sum _{k=0}^{m-1}\frac{v_{2}^{(k)}({\zeta },0)}{{s}^{2-{{\varrho }}+k}} +{A}\Bigg [\frac{\partial ^2 v_{2}(\zeta ,{\Im })}{\partial \zeta ^2}+2v_{2}(\zeta ,{\Im })\frac{\partial v_{2}(\zeta ,{\Im })}{\partial \zeta }-v_{2}(\zeta ,{\Im })\frac{\partial v_{1}(\zeta ,{\Im })}{\partial \zeta }\\ &\quad\quad\quad\quad\quad\quad\quad\;-v_{1}(\zeta ,{\Im })\frac{\partial v_{2}(\zeta ,{\Im })}{\partial \zeta }\Bigg ]\Bigg )\Bigg ]\\ \end{aligned} \end{aligned}$$

The following formula is obtained by using the Aboodh transform iteratively:

$$\begin{aligned} \begin{aligned}{}&(v_1)_{0}(\zeta , {\Im })={A}^{-1}\left[ \frac{1}{{s}^{{{\varrho }}}}\left( \sum _{k=0}^{m-1}\frac{v_{1}^{(k)}({\zeta },0)}{{s}^{2-{{\varrho }}+k}}\right) \right] \\&\quad \quad \quad \quad \quad ={A}^{-1}\Big [\frac{v_{1}({\zeta },0)}{{s}^2}\Big ]\\&\quad \quad \quad \quad \quad =\sin (\zeta ), \end{aligned} \\ \begin{aligned}{}&(v_2)_{0}(\zeta , {\Im })={A}^{-1}\left[ \frac{1}{{s}^{{{\varrho }}}}\left( \sum _{k=0}^{m-1}\frac{v_{2}^{(k)}({\zeta },0)}{{s}^{2-{{\varrho }}+k}}\right) \right] \\&\quad \quad \quad \quad \quad ={A}^{-1}\Big [\frac{v_{2}({\zeta },0)}{{s}^2}\Big ]\\&\quad \quad \quad \quad \quad =\sin (\zeta ), \end{aligned} \end{aligned}$$

By using the RL integral on Eqs 92 and 93, we get the corresponding form.

100 $$\begin{aligned} \begin{aligned}{}&v_1(\zeta , {\Im })=\sin (\zeta ) +{A}\Bigg [\frac{\partial ^2 v_{1}(\zeta ,{\Im })}{\partial \zeta ^2}+2v_{1}(\zeta ,{\Im })\frac{\partial v_{1}(\zeta ,{\Im })}{\partial \zeta }-v_{2}(\zeta ,{\Im })\frac{\partial v_{1}(\zeta ,{\Im })}{\partial \zeta }-v_{1}(\zeta ,{\Im })\frac{\partial v_{2}(\zeta ,{\Im })}{\partial \zeta }\Bigg ]\\ \end{aligned} \end{aligned}$$

101 $$\begin{aligned} \begin{aligned}{}&v_2(\zeta , {\Im })=\sin (\zeta ) +{A}\Bigg [\frac{\partial ^2 v_{2}(\zeta ,{\Im })}{\partial \zeta ^2}+2v_{2}(\zeta ,{\Im })\frac{\partial v_{2}(\zeta ,{\Im })}{\partial \zeta }-v_{2}(\zeta ,{\Im })\frac{\partial v_{1}(\zeta ,{\Im })}{\partial \zeta }-v_{1}(\zeta ,{\Im })\frac{\partial v_{2}(\zeta ,{\Im })}{\partial \zeta }\Bigg ]\\ \end{aligned} \end{aligned}$$

These are the few terms that are produced by the NITM process.

102 $$\begin{aligned} \begin{aligned}{}&{v_{1}}_{0}(\zeta ,{\Im })=\sin (\zeta ),\\&{v_{2}}_{0}(\zeta ,{\Im })=\sin (\zeta ),\\&{v_{1}}_{1}(\zeta ,{\Im })=-\frac{{\Im }^{{\varrho }}\sin (\zeta )}{\Gamma (p+1)},\\&{v_{2}}_{1}(\zeta ,{\Im })=-\frac{{\Im }^{{\varrho }}\sin (\zeta )}{\Gamma (p+1)},\\&{v_{1}}_{2}(\zeta ,{\Im })=\frac{{\Im }^{2p}\sin (\zeta )}{\Gamma (2p+1)},\\&{v_{2}}_{2}(\zeta ,{\Im })=\frac{{\Im }^{2p}\sin (\zeta )}{\Gamma (2p+1)}.\\&{v_{1}}_{3}(\zeta ,{\Im })=-\frac{{\Im }^{3p}\sin (\zeta )}{\Gamma (3p+1)},\\&{v_{2}}_{3}(\zeta ,{\Im })=-\frac{{\Im }^{3p}\sin (\zeta )}{\Gamma (3p+1)}.\\ \end{aligned} \end{aligned}$$

The NITM algorithm’s final result is as under

103 $$\begin{aligned} \begin{aligned} v_{1}(\zeta ,{\Im })={v_{1}}_{0}(\zeta ,{\Im })+{v_{1}}_{1}(\zeta ,{\Im })+{v_{1}}_{2}(\zeta ,{\Im })+\cdots . \end{aligned} \end{aligned}$$

104 $$\begin{aligned} \begin{aligned} v_{2}(\zeta ,{\Im })={v_{2}}_{0}(\zeta ,{\Im })+{v_{2}}_{1}(\zeta ,{\Im })+{v_{2}}_{2}(\zeta ,{\Im })+\cdots . \end{aligned} \end{aligned}$$

105 $$\begin{aligned} \begin{aligned}{}&v_1(\zeta ,t)=\sin (\zeta )+\frac{\sin (\zeta ) {\Im } ^{2 p}}{\Gamma (2 p+1)}-\frac{\sin (\zeta ) {\Im } ^{3 p}}{\Gamma (3 p+1)}-\frac{\sin (\zeta ) {\Im } ^p}{\Gamma (p+1)}+\cdots .\\ \end{aligned} \end{aligned}$$

106 $$\begin{aligned} \begin{aligned}{}&v_2(\zeta ,t)=\sin (\zeta )+\frac{\sin (\zeta ) {\Im } ^{2 p}}{\Gamma (2 p+1)}-\frac{\sin (\zeta ) {\Im } ^{3 p}}{\Gamma (3 p+1)}-\frac{\sin (\zeta ) {\Im } ^p}{\Gamma (p+1)}+\cdots .\\ \end{aligned} \end{aligned}$$

**Figure 7:**
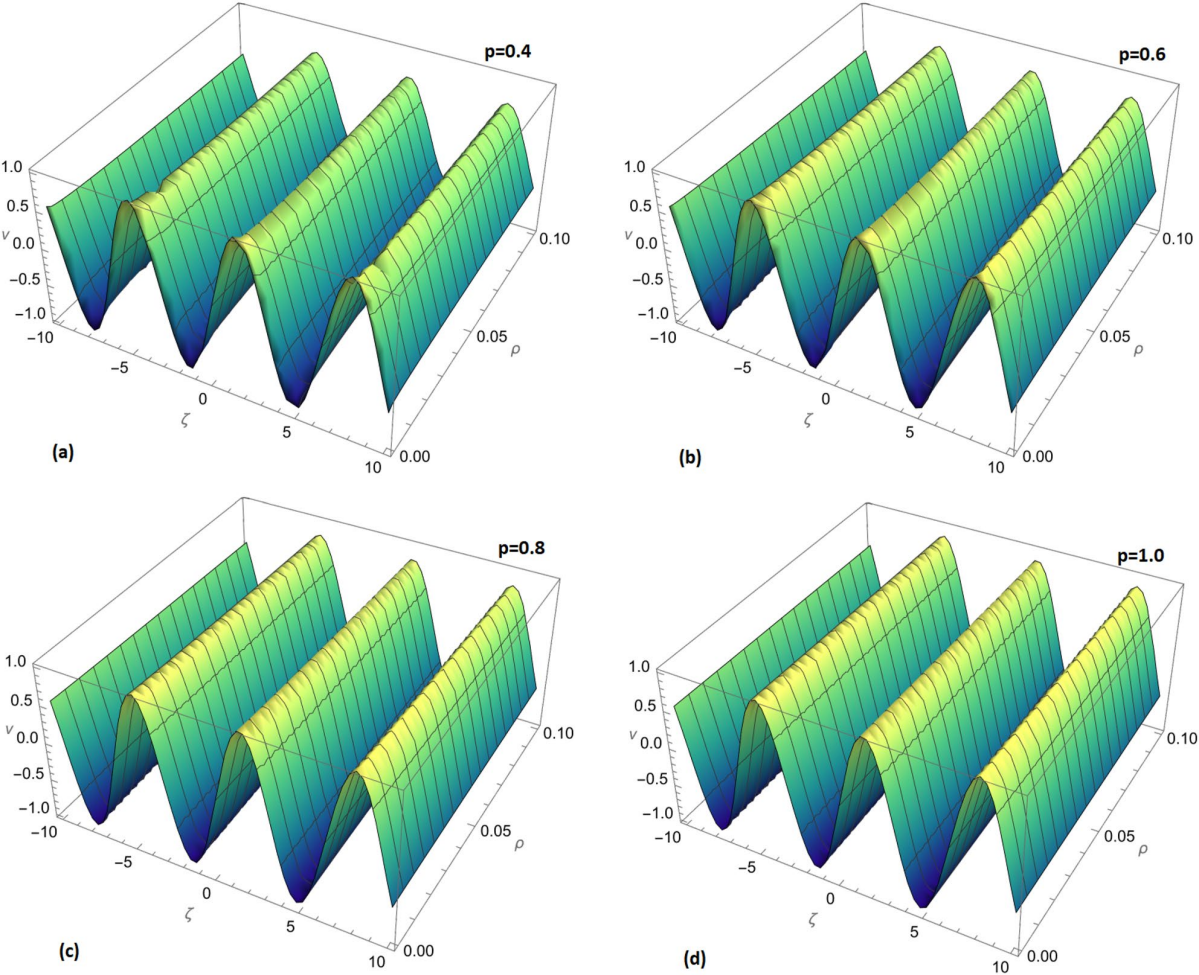
Fractional order comparison of $v_{1}(\zeta ,{\Im })$ and $v_{2}(\zeta ,{\Im })$ for ${\Im }=0.1$.

**Figure 8:**
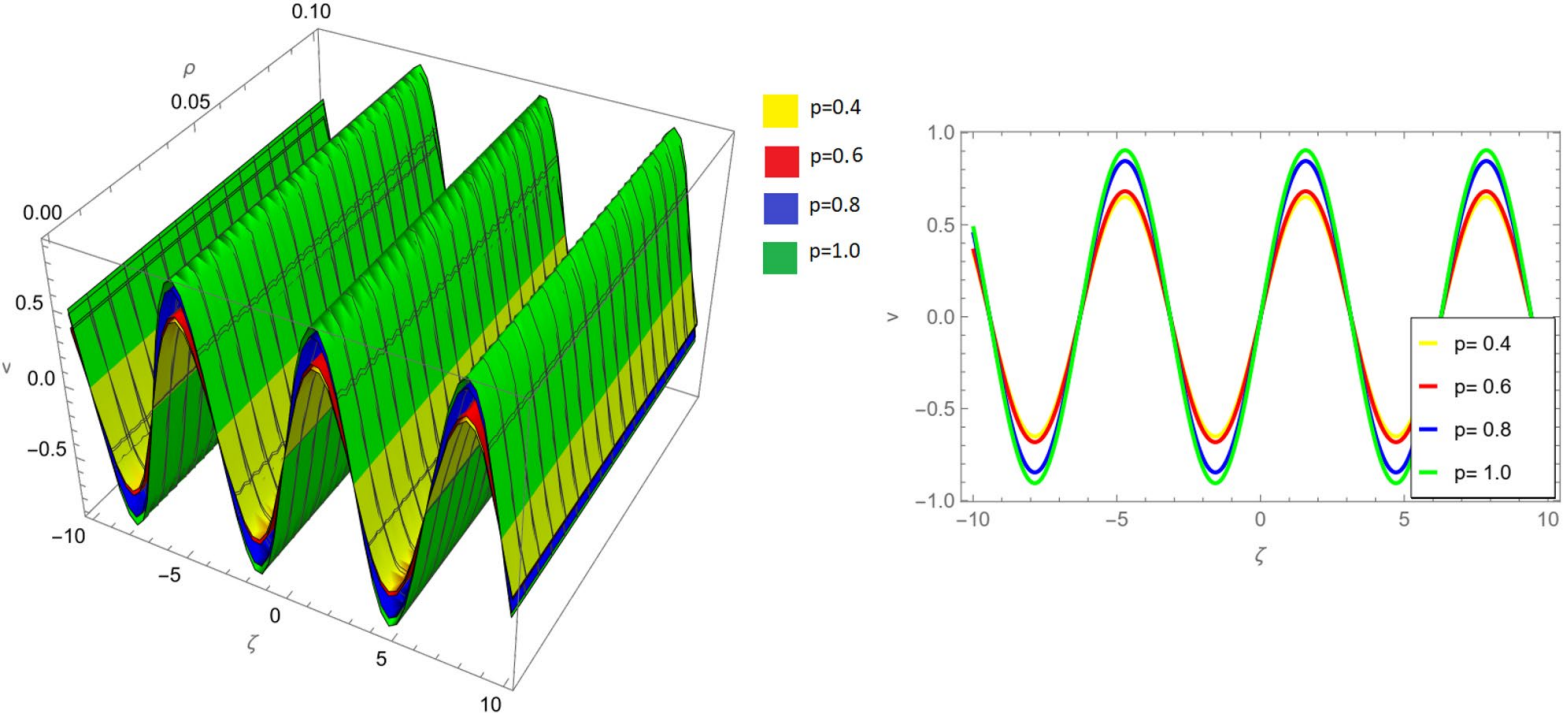
Fractional order 3D and 2D comparison of $v_{1}(\zeta ,{\Im })$ and $v_{2}(\zeta ,{\Im })$ for ${\Im }=0.1$.

**Table 4:** Analysis of the NITM solution for various fractional order of example 2 for $v_{1}(\zeta ,{\Im })$ and $v_{2}(\zeta ,{\Im })$ for ${\Im }=0.1$.

$\zeta$	$NITM_{P=0.6}$	$NITM_{p=0.8}$	$NITM_{P=1.0}$	Exact	Error of $p=0.6$	Error of $p=0.8$	Error of $p=1.0$
− 0.5	− 0.36821	− 0.405666	− 0.433802	− 0.433802	0.065592	0.0281359	3.929565 $\times\,10^{-7}$
− 0.4	− 0.299082	− 0.329507	− 0.35236	− 0.35236	0.0532778	0.0228537	3.191830 $\times\,10^{-7}$
− 0.3	− 0.226967	− 0.250055	− 0.267398	− 0.267398	0.0404312	0.0173431	2.422203 $\times\,10^{-7}$
− 0.2	− 0.152583	− 0.168104	− 0.179763	− 0.179763	0.0271807	0.0116592	1.628374 $\times\,10^{-7}$
− 0.1	− 0.0766744	− 0.0844741	− 0.090333	− 0.090333	0.0136586	0.00585889	8.182750 $\times\,10^{-8}$
0.1	0.0766744	0.0844741	0.090333	0.090333	0.0136586	0.00585889	8.182750 $\times\,10^{-8}$
0.2	0.152583	0.168104	0.179763	0.179763	0.0271807	0.0116592	1.628374 $\times\,10^{-7}$
0.3	0.226967	0.250055	0.267398	0.267398	0.0404312	0.0173431	2.422203 $\times\,10^{-7}$
0.4	0.299082	0.329507	0.35236	0.35236	0.0532778	0.0228537	3.191830 $\times\,10^{-7}$
0.5	0.36821	0.405666	0.433802	0.433802	0.065592	0.0281359	3.929565 $\times\,10^{-7}$

**Table 5:** Example 1 error comparison for both methods.

$\zeta$	*Exact*	$ARPSM_P=1$	$NITM_P=1$	$Error_{ARPSM}$	$Error_{NITM}$
0.1	0.223549	0.223529	0.223549	0.0000195862	2.886387 $\times\,10^{-12}$
0.2	0.223379	0.223343	0.223379	0.0000362061	5.6020329 $\times\,10^{-12}$
0.3	0.223098	0.223045	0.223098	0.0000526159	8.2351522 $\times\,10^{-12}$
0.4	0.222707	0.222638	0.222707	0.0000687278	1.0747543 $\times\,10^{-11}$
0.5	0.222206	0.222121	0.222206	0.0000844573	1.3103146 $\times\,10^{-11}$
0.6	0.221596	0.221497	0.221596	0.0000997242	1.5269125 $\times\,10^{-11}$
0.7	0.22088	0.220766	0.22088	0.000114453	1.7216318 $\times\,10^{-11}$
0.8	0.220059	0.21993	0.220059	0.000128575	1.8919520 $\times\,10^{-11}$
0.9	0.219135	0.218993	0.219135	0.000142027	2.0358186 $\times\,10^{-11}$
1.	0.21811	0.217955	0.21811	0.000154751	2.1516441 $\times\,10^{-11}$

**Table 6:** Example 2 error comparison for both methods.

/$\zeta$	Exact	$ARPSM_P=1$	$NITM_P=1$	$Error_{ARPSM}$	$Error_{NITM}$
− 0.5	− 0.433802	− 0.4338	− 0.433802	1.958310 $\times\,10^{-6}$	3.929565 $\times\,10^{-7}$
− 0.4	− 0.35236	− 0.352359	− 0.35236	1.590658 $\times\,10^{-6}$	3.191830 $\times\,10^{-7}$
− 0.3	− 0.267398	− 0.267397	− 0.267398	1.207112 $\times\,10^{-6}$	2.422203 $\times\,10^{-7}$
− 0.2	− 0.179763	− 0.179763	− 0.179763	8.115051 $\times\,10^{-7}$	1.628374 $\times\,10^{-7}$
− 0.1	− 0.090333	− 0.0903326	− 0.090333	4.077898 $\times\,10^{-7}$	8.182750 $\times\,10^{-8}$
0.1	0.090333	0.0903326	0.090333	4.077898 $\times\,10^{-7}$	8.182750 $\times\,10^{-8}$
0.2	0.179763	0.179763	0.179763	8.115051 $\times\,10^{-7}$	1.628374 $\times\,10^{-7}$
0.3	0.267398	0.267397	0.267398	1.207112 $\times\,10^{-6}$	2.422203 $\times\,10^{-7}$
0.4	0.35236	0.352359	0.35236	1.590658 $\times\,10^{-6}$	3.191830 $\times\,10^{-7}$
0.5	0.433802	0.4338	0.433802	1.958310 $\times\,10^{-6}$	3.929565 $\times\,10^{-7}$

Table 3 provides a comprehensive analysis of the NITM solution for various fractional orders in Example 1, specifically for $v_{1}(\zeta ,{\Im })$ when ${\Im }=0.1$. The fractional order plays a crucial role in determining the accuracy and behavior of the solution, and the table offers insights into its impact.

In Fig. 5, a visual comparison is presented between the exact solution and the approximate solution of $v_{1}(\zeta ,{\Im })$ for ${\Im }=0.1$. This graphical representation allows for a clear assessment of the agreement between the two solutions.

Figure 6 further enhances the comparison by providing a detailed view. Subfigure (a) displays the overall comparison between the exact and approximate solutions, while subfigure (b) focuses on the fractional order comparison of $v_{1}(\zeta ,{\Im })$ for ${\Im }=0.1$. These visualizations aid in understanding how the fractional order influences the solution’s accuracy.

Figure 7 extends the analysis to a fractional order comparison of both $v_{1}(\zeta ,{\Im })$ and $v_{2}(\zeta ,{\Im })$ for ${\Im }=0.1$. The fractional order plays a significant role in shaping the solutions of both variables, and this figure provides a comprehensive view of their behavior.

Figure 8 offers a more detailed perspective, presenting fractional order 3D and 2D comparisons of $v_{1}(\zeta ,{\Im })$ and $v_{2}(\zeta ,{\Im })$ for ${\Im }=0.1$. These visualizations facilitate a deeper understanding of the solutions’ characteristics in different dimensions.

Table 4 extends the analysis to Example 2, providing a detailed examination of the NITM solution for various fractional orders for both $v_{1}(\zeta ,{\Im })$ and $v_{2}(\zeta ,{\Im })$ for ${\Im }=0.1$.

Tables 5 and 6 offer error comparisons for both methods in Example 1 and Example 2, respectively. These tables quantify the differences between the exact and approximate solutions, providing valuable insights into the performance of the NITM method in different scenarios.

## Conclusion

In conclusion, this work has proved the efficiency of the Aboodh residual power series method (ARPSM) and the Aboodh transform iteration method (ATIM) to uncouple the Modified Korteweg-de Vries equation (mKdV) and coupled Burger’s equations under the Caputo operator. The application of these methods has proven instrumental in obtaining analytical solutions for these nonlinear differential equations. Through this investigation, valuable insights into the behavior and dynamics of the studied equations within a fractional calculus framework have been revealed. This numerical approach furnishes different solutions in scope of the mKdV equation and coupled Burger’s equations, as well as other nonlinear problems, leading to deeper understanding of nonlinearity in various physics systems and aids in a better model making. We saw in the figures and tables discussion that the graphs obtained to find the solutions through the use of ARPSM and ATIM methods for various parameters and fractional orders the method predicted the dynamics of the mKdV equation and coupled Burger’s equations well. Furthermore, as the results from experiments shown in tables are analyzed in a good way, numerically convergence and error analyses are done, which give the results of ARPSM and ATIM techniques as useful. This research adds to the knowledge of nonlinear wave dynamics and applications in fractional calculus resulting in the excellent toolbox for investigating wave phenomena in the bed of sciences and engineering. Further investigations of wave nonlinear phenomena could uncover new nonlinear wave equations and broaden the scope of the developed methods to study other complicated systems, ultimately leading to better capabilities in designing better models for wave behavior predictions.

## Data Availability

The data sets used and/or analysed during the current study available from the corresponding author on reasonable request.
